# Vesicles Bearing *Toxoplasma* Apicoplast Membrane Proteins Persist Following Loss of the Relict Plastid or Golgi Body Disruption

**DOI:** 10.1371/journal.pone.0112096

**Published:** 2014-11-04

**Authors:** Anne Bouchut, Jennifer A. Geiger, Amy E. DeRocher, Marilyn Parsons

**Affiliations:** 1 Seattle Biomedical Research Institute, Seattle, WA, United States of America; 2 Dept. of Global Health, University of Washington, Seattle, WA, United States of America; University of Melbourne, Australia

## Abstract

*Toxoplasma gondii* and malaria parasites contain a unique and essential relict plastid called the apicoplast. Most apicoplast proteins are encoded in the nucleus and are transported to the organelle *via* the endoplasmic reticulum (ER). Three trafficking routes have been proposed for apicoplast membrane proteins: (i) vesicular trafficking from the ER to the Golgi and then to the apicoplast, (ii) contiguity between the ER membrane and the apicoplast allowing direct flow of proteins, and (iii) vesicular transport directly from the ER to the apicoplast. Previously, we identified a set of membrane proteins of the *T. gondii* apicoplast which were also detected in large vesicles near the organelle. Data presented here show that the large vesicles bearing apicoplast membrane proteins are not the major carriers of luminal proteins. The vesicles continue to appear in parasites which have lost their plastid due to mis-segregation, indicating that the vesicles are not derived from the apicoplast. To test for a role of the Golgi body in vesicle formation, parasites were treated with brefeldin A or transiently transfected with a dominant-negative mutant of Sar1, a GTPase required for ER to Golgi trafficking. The immunofluorescence patterns showed little change. These findings were confirmed using stable transfectants, which expressed the toxic dominant-negative sar1 following Cre-*loxP* mediated promoter juxtaposition. Our data support the hypothesis that the large vesicles do not mediate the trafficking of luminal proteins to the apicoplast. The results further show that the large vesicles bearing apicoplast membrane proteins continue to be observed in the absence of Golgi and plastid function. These data raise the possibility that the apicoplast proteome is generated by two novel ER to plastid trafficking pathways, plus the small set of proteins encoded by the apicoplast genome.

## Introduction

*Toxoplasma gondii* is an obligate intracellular protozoan parasite belonging to the phylum Apicomplexa, which also includes the malaria parasite *Plasmodium falciparum*. *T. gondii*, one of the most successful known parasites, infects one-third of the world's human population and its ability to differentiate into quiescent bradyzoite cysts leads to lifelong persistence [1], [2]. While infection of immunocompetent hosts is often asymptomatic, *T. gondii* has been recognized as a major pathogen of immunocompromised patients, *i.e.* transplant recipients or those with HIV/AIDS, as well as being vertically transmitted to the fetus from recently infected mothers. Indeed, *T. gondii* is the causative agent of both toxoplasmic encephalitis, the most common cause of focal brain lesions in people with HIV/AIDS, and congenital toxoplasmosis, a leading cause of neurological birth defects in children. Insights leading to new therapeutic options are needed since available drugs can have serious side effects.

Among the characteristics of many Apicomplexa is the presence of a unique organelle, the apicoplast. It is a non-photosynthetic plastid acquired by secondary endosymbiosis from an alga, i.e., a secondary plastid. The apicoplast is the site of several anabolic pathways including iron-sulfur cluster biosynthesis, lipoic acid synthesis [3], part of the heme biosynthesis pathway [4], type II fatty acid synthesis [5], and the non-mevalonate pathway of isoprenoid biosynthesis [6], [7]. The last pathway is found in apicoplasts of all species, is essential to the parasites (although not necessarily in all developmental stages), and is absent from animal hosts. Interference with apicoplast DNA replication [8] or translation [9], [10], as well as inhibition of certain apicoplast metabolic functions [5], [6], is lethal for *T. gondii* and *Plasmodium*. Thus the organelle is a potential target for the development of novel drugs.

The apicoplast's small genome (35 kb) encodes primarily RNAs and proteins important for the propagation of the organelle. Hence most plastid functions are fulfilled by numerous nucleus-encoded proteins. These are typically imported into the apicoplast lumen by virtue of an N-terminal bipartite sequence which is composed of a signal peptide and an adjacent transit peptide (signal+transit, S+T). Proteins destined for the lumen of the apicoplast, such as acyl carrier protein (ACP), are first imported into the endoplasmic reticulum (ER), where the signal sequence is cleaved and then transferred to the apicoplast *via* an unknown mechanism. There the transit peptide is removed. Several lines of evidence indicate that targeting of apicoplast luminal proteins bypasses the Golgi body. First, it is resistant to the Golgi inhibitor brefeldin A (BFA, which blocks COPI coat assembly leading to collapse of Golgi body to the ER) [11], [12]. Second, targeting is resistant to low temperature treatment which disrupts ER to Golgi trafficking [11]. Finally, apicoplast luminal proteins bearing a sequence that mediates retrieval of proteins from the Golgi back to the ER were still localized to the apicoplast, indicating that the protein did not encounter the Golgi-localized retrieval receptor [11], [12].

The apicoplast is a product of secondary endosymbiosis and, as such, is surrounded by four membranes. The inner two are presumably homologous to chloroplast membranes; the third or periplastid membrane likely originated from the algal plasma membrane and the outer membrane is thought to have arisen from an endocytic membrane from the apicomplexan progenitor. Proteins residing in the membranes or intermembrane spaces of the apicoplast appear to fall into two categories: those that possess S+T targeting sequences and those that do not. Examples of those which bear a S+T sequence include Tic20 [13] and Tic22 [14], [15] (components of the import apparatus) and PfiTPT (a transporter of the *P. falciparum* apicoplast innermost membrane) [16], as well as some proteins of the periplastid space or membrane such as components related to the ER-associated degradation machinery (e.g., Ufd1 and Der1-related proteins) [14], [17]–[19]. Those that lack a canonical targeting sequence include two non-luminal proteins recently identified in a systematic screen [20], as well as three membrane-associated proteins that have been examined in some detail: the protease FtsH1 [21], [22], the thioredoxin ATrx1 [23], and the transporter APT1 [24], [25]. FtsH1 is a 1250 aa protease with a single transmembrane domain that is processed at both N- and C-termini. ATrx1 is a peripheral membrane protein that possesses a signal anchor sequence and is N-terminally processed, removing the anchor. In contrast, APT1 is a polytopic membrane protein that undergoes no detectable processing. In addition to being present at the apicoplast, immunoelectron microscopy has detected each of these proteins in large vesicles (V^ap^), which are recognized as apparent vesicles and tubules in immunofluorescence analysis (IFA) [22]–[24]. V^ap^ are not artifacts of overexpression since they can be seen in untransfected cells using a monoclonal antibody (mAb) to ATrx1 [23]. The apicoplast division cycle has been described by Streipen et al. [26]. In stage 1, the round plastid is associated with the centriole (early G1). In stage 2, the plastid becomes ovoid, remaining associated with the now-duplicated centrioles. In stage 3, it elongates and approaches the nucleus (late G1 to S), forming a U-shape in stage 4. By stage 5, the apicoplast has divided and mitosis is underway, and at stage 6, mitosis is complete. In stages 2–4 of the apicoplast division cycle as described, V^ap^ become prominent. In some immunoelectron microscopy images, V^ap^ are observed very close to or merging with the apicoplast [23]. Others have shown that V^ap^ and the apicoplast bear phosphatidylinositol 3-phosphate (PI3P) and that overexpression of a PI3P binding protein leads to loss of the apicoplast [27]. Additionally, mutations in APT1 that block targeting to the apicoplast also block APT1 recruitment into V^ap^. Based on these findings, we [24] and others [27] have suggested that V^ap^ are transport vesicles, which serve to move at least a subset of apicoplast membrane proteins to the organelle. However, it is possible that the V^ap^ represent degradation intermediates originating from the apicoplast or persistent structures originating from the ER or Golgi body. Here, we will refer to this group of apicoplast proteins found abundantly on V^ap^ as ApV proteins.

Three hypothetical routes for ApV protein transport to the apicoplast have been proposed: (i) transit in vesicles first to the Golgi body and then to the apicoplast, following the typical route for secretory proteins; (ii) direct flow from the ER to the apicoplast *via* continuous membrane; (iii) transport in vesicles directly from the ER to the apicoplast. Here we probe the relationship of the Golgi body and V^ap^ pharmacologically using BFA and genetically *via* expression of a dominant-negative mutant of SAR1, the GTPase that initiates ER to Golgi trafficking. V^ap^ persisted despite these treatments. These studies suggest that recruitment of ApV proteins into V^ap^ may be Golgi-independent. Additionally our studies show that luminal proteins are largely absent from V^ap^.

## Results

### A luminal marker protein does not traffic via V^ap^

**Figure 1:**
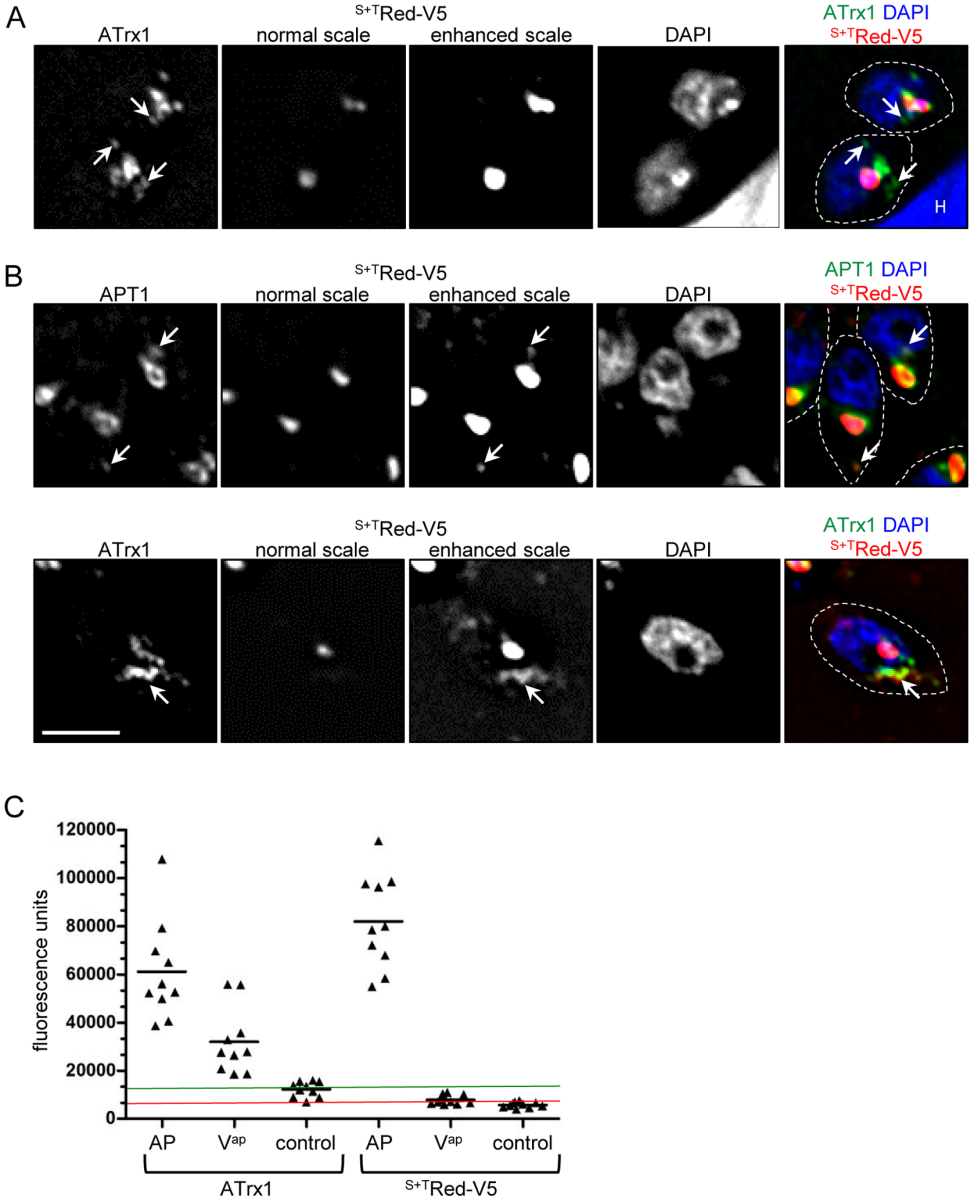
V^ap^ are not major vehicles for luminal protein trafficking to the apicoplast. For IFA analysis here and elsewhere unless indicated, proteins were detected by mAbs directed against epitope tags followed by fluorochrome-coupled secondary antibodies as described in Methods. In this case, the apicoplast membrane proteins were detected anti-HA mAb was followed by FITC-coupled secondary antibodies and ^S+T^Red-V5 was detected by anti-V5 mAb followed by Texas Red-coupled antibodies to bypass the need for maturation of the HcRed chromophore. Here, as in other figures, the color coding for merged images is indicated by the text color above the merged images, while dashed lines mark the outline of the parasite. In this experiment, the parasites co-expressed ^S+T^Red-V5 driven by the *ACP* promoter and epitope-tagged ApV proteins APT1-HA or ATrx1-HA. A) IFA showing the pattern seen in about 80% of parasites with ATrx1-HA in V^ap^ (arrows) near the apicoplast. One set of anti-V5 images is scaled normally and the second is scaled to detect fainter signals (the staining at the apicoplast is then saturated). No evidence of localization of the luminal marker ^S+T^Red-V5 with V^ap^ was observed when scanning through the deconvolved planes. “H” marks a host cell nucleus. B) In the approximately 20% parasites with evident V^ap^, occasional regions staining for membrane-associated proteins (V^ap^, arrows) also showed a weak signal for the luminal marker ^S+T^Red-V5. Bar, 2 µm. C) Individual parasites with V^ap^ as detected by the presence of ATrx1-HA were randomly chosen for quantitation of ATrx1-HA and ^S+T^Red-V5 signals. The average fluorescence corresponding to each protein in a 90 pixel area covering either the apicoplast (AP), vesicles (V^ap^) or adjacent regions (control) was determined and plotted for each individual parasite (see Methods). The mean florescence signal seen in the parasite population is marked for each region analyzed (black lines). The raw fluorescence intensities for the adjacent regions averaged 12243 fluorescence units for ATrx1-HA and 5785 for ^S+T^Red-V5, very close to the average background of 12267 (anti-HA, green line) and 6577 (anti-V5, red line) for these channels in untransfected RH parasites on the same slide.

^S+T^Red-V5 was prominently observed at the apicoplast as a dot surrounded by the membrane proteins or in an elongated bead and tubule appearance interspersed with the membrane proteins, as previously detailed [24]. When we rescaled the signal to search for dimly stained objects, no additional structures were revealed in 80% of the parasites (Fig. 1A). In 20% of parasites bearing V^ap^ (Fig. 1B) we observed faint perinuclear ^S+T^Red-V5 staining, characteristic of the ER, suggesting the presence of newly synthesized ^S+T^Red-V5. Commensurate with the ER staining, this population was enriched for parasites earlier in the apicoplast division cycle when plastid proteins are beginning to be synthesized, since almost all were stage 1 or stage 2 (>95%). These cells had occasional spots of slightly more concentrated fluorescence signal and most of these coincided with foci staining for APT1 or ATrx1. However, the converse was not true: the majority of APT1^+^ or ATrx1^+^ V^ap^ within these same cells did not co-stain for the luminal protein.

When expressed from their cognate promoters, FtsH1, ATrx1 and APT1 [22]–[24] have been observed in V^ap^, large electron dense vesicles near the apicoplast, suggesting that these proteins may travel to the apicoplast *via* a vesicle trafficking pathway. In contrast, previous studies have not revealed vesicles bearing luminal proteins. However, most of these studies employed heterologous promoters with different timing of expression, raising the possibility that some normal trafficking pathways might not be readily detected. We hypothesized that luminal S+T bearing proteins may travel to the plastid in V^ap^, and that these trafficking intermediates could be detected when expression was driven by a promoter of a gene encoding an apicoplast luminal protein (these genes are coordinately expressed along with genes encoding apicoplast membrane proteins [28]). For these studies we chose the *ACP* promoter for the luminal marker. It drives expression with the same temporal pattern as *APT1*, although the *ACP* mRNA expression level is about 4-fold higher (Fig. S1) ([28] and ToxoDB). The *ATrx1* mRNA shows two closely spaced peaks, the second of which corresponds to peak expression of *ACP* and *APT1*. We carefully examined the staining pattern of a luminally targeted *Heteractis crispa* red fluorescent protein tagged with V5 epitopes (^S+T^Red-V5) (Fig. 1). We used antibodies to the V5 epitope to enhance the signal as compared to intrinsic fluorescence and to detect protein that had not yet matured to its fluorescent form [29]. V^ap^ were revealed by detection of epitope-tagged APT1 or ATrx1 expressed using their cognate promoters (see Methods for description of tagged proteins). The populations examined contained a mix of parasites at different stages of the apicoplast division cycle ensuring detection of co-trafficking, should it exist (for example in the analysis of APT1-HA and ^S+T^Red-V5, the populations averaged: 30% stage 1+2, 13% stage 3+4 and 7% stage 5+6).

We quantified our observations by examining the average signal of the luminal marker protein ^S+T^Red-V5 and the ApV protein ATrx1 at the apicoplast, at V^ap^ (as defined by ATrx1), and at adjacent non-apicoplast control regions (Fig. 1C). Background signals in each channel were determined by assessing average fluorescence in co-cultured wild type RH parasites (see Materials and Methods). We determined that the average ATrx1 and ^S+T^Red-V5 signals at the apicoplast were 5 and 12.4 times that of the RH background respectively while the signals for adjacent regions were very close to background (1.0 and 0.9 times background respectively). The mean ATrx1 signal corresponding to V^ap^ was about 50% of that seen at the apicoplast. In contrast, in those same regions, the ^S+T^Red-V5 signal represented a much lower fraction of that seen at the apicoplast (10%), hovering at the background level. This difference is not an artifact related to differential stability of the two proteins, as pulse-chase analysis showed they have similar half-lives (Fig. S2). These results suggest that even though integral and peripheral membrane proteins can sometimes co-localize outside the apicoplast to apparent V^ap^, V^ap^ are not the main mode of transit for the luminal marker protein ^S+T^Red-V5. This work does not rule out the trafficking of apicoplast proteins via small vesicles which would not be resolved by deconvolution microscopy and could be spread over a large area of the cell. If such vesicles are short-lived (rapidly fuse with their destination membrane), it is doubtful that they would be detected as a significant signal even as a “fuzz” above background. Our previous findings showed that trafficking ^S+T^Red-V5 from the ER to the apicoplast was rapid, being mostly complete by 10 minutes [11]. This inability to detect luminal proteins contrasts with the ready detection of other proteins of the non-luminal apicoplast compartments in V^ap^ [22], [23]. For example, Tic22, a protein of the innermost intermembrane space co-localized with ATrx1 in these structures, as did Der1-ap, a protein of the periplastid membrane (Fig. S3).

**Figure 2:**
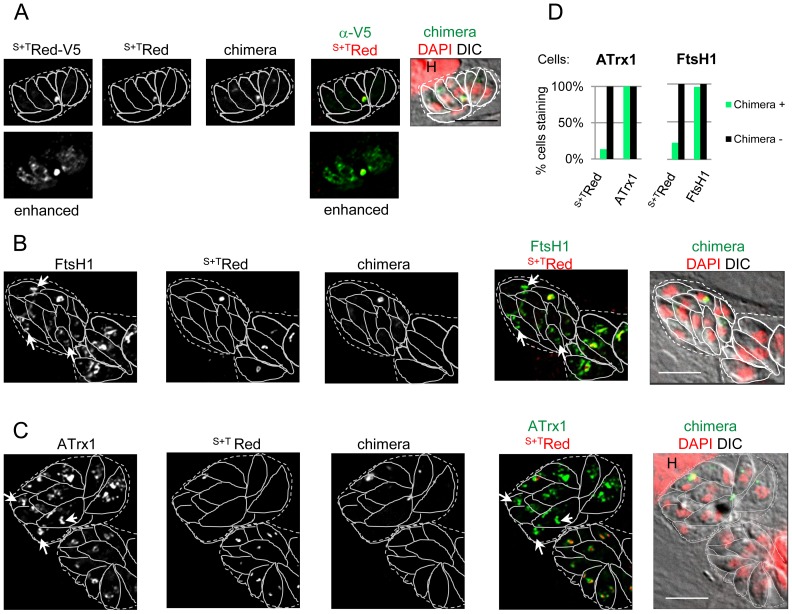
V^ap^ persist in parasites with plastid loss. *T. gondii* expressing the indicated tagged apicoplast proteins were transiently transfected with a plasmid encoding ^S+T^YFP-ROP1 (chimera, endogenous fluorescence) to induce plastid mis-segregation. After 40 hours to allow for apicoplast loss through several cell divisions, the samples were subjected to IFA. Vacuoles with one or more parasites expressing the chimeric protein were analyzed. Individual cells and vacuoles are outlined with solid lines and dashed lines respectively. The markers are indicated above each panel. DIC, differential interference contrast, H indicates host cell nucleus. A) Loss of luminal marker in parasites expressing the “poison” chimera. ^S+T^Red-V5 was detected with both anti-V5 mAb (followed by secondary antibody coupled to Dylight 649; panels labeled ^S+T^Red-V5), and through intrinsic fluorescence (panels here and in B, C labeled ^S+T^Red). The lower panels show enhanced scaling of ^S+T^Red-V5 detected with anti-V5 to highlight faint ER-like staining. Bar = 5 µM. B) Continued formation of V^ap^ bearing FtsH1. FtsH1/^S+T^Red parasites were transiently transfected with the chimeric construct (detected by endogenous fluorescence) and FtsH1 was detected with anti-V5 mAb (followed by secondary antibody coupled to DyLight 649). Background of the ^S+T^Red images in panels B and C were adjusted to correct for crossover fluorescence from the DyLight 649 fluorophore. Arrows indicate V^ap^-like staining in cells lacking an apicoplast. Vacuoles bearing transfected parasites (upper left) and untransfected parasites (lower right) are shown. Bar = 5 µM. C) Continued formation of V^ap^ bearing ATrx1. ATrx1/^S+T^Red expressing cells were transiently transfected with ^S+T^ROP1-YFP, which was detected by endogenous fluorescence. ATrx1 was detected with anti-HA mAb coupled to DyLight 649. Arrows indicate V^ap^-like staining in cells lacking an apicoplast. Vacuoles bearing transfected parasites (upper left) and untransfected parasites (lower right) are shown. Parasites in the lower vacuole are in stage 1 of the organelle division cycle and therefore have few V^ap^. Bar = 5 µM. D) Quantitation of V^ap^ in apicoplast-deficient parasites. ATrx1-4HA/^S+T^Red and FtsH1-V5^233^-HA/^S+T^Red expressing cell lines were transiently transfected with the chimeric ^S+T^YFP-ROP1 construct and vacuoles were scored for the presence or absence of YFP in at least one parasite (indicating expression of the chimeric protein in the original invading parasite). Individual parasites within each vacuole were then scored for the presence or absence of the luminal protein ^S+T^Red (detected by endogenous fluorescence) and the apicoplast membrane protein (detected by anti-HA or anti-V5 mAbs followed by anti-mouse IgG coupled to DyLight 649). The bar graph plots the percentage of cells bearing each marker protein in vacuoles derived from transfected (chimera^+^) and untransfected (chimera^−^) parasites. In the ATrx1 sample, 96 chimera^+^ and 128 chimera^−^ cells were counted; in the FtsH1 sample, 27 chimera^+^ and 48 chimera^−^ cells were counted. These results are representative of three independent experiments.

To further compare ApV proteins to luminal proteins, we examined their localization in *T. gondii* lacking an apicoplast. These parasites were generated by using a “poison” construct described by He et al. [30], which encodes a chimeric protein composed of an apicoplast targeting sequence fused to YFP followed by the mature domain of the rhoptry protein Rop1 (^S+T^YFP-ROP1). In previous studies, it was shown that the chimeric protein targets to the apicoplast and disrupts plastid segregation, often resulting in parasitophorous vacuoles containing one cell with a large plastid and several cells apparently lacking apicoplast luminal proteins as well as the apicoplast genome [30]. The plasmid was transiently transfected into cells expressing a red fluorescent luminal protein marker (^S+T^Red or ^S+T^Red-V5) along with tagged ApV proteins ATrx1 or FtsH1. Our analysis focused on those vacuoles with strong expression of the chimeric protein in only one parasite. There was a marked difference in the fate of the luminal and ApV proteins in cells lacking an apicoplast (Fig. 2). As expected, the apicoplast luminal marker partitioned with the chimeric protein and these were either localized together typically at the apicoplast (but occasionally at the residual body), or not detected at all by intrinsic fluorescence. Using anti-V5 antibody to visualize ^S+T^Red-V5 prior to chromophore maturation additionally revealed the protein in a faint ER-like pattern in some cells (Fig. 2A, “enhanced”), suggesting continued ^S+T^Red-V5 production. This pattern appeared to be somewhat more frequent in parasites that lacked an apicoplast, although the difference from control was not statistically significant. ATrx1 and FtsH1 on the other hand accumulated in structures apical to the nucleus (examples indicated by arrows), similar to the V^ap^ seen in the cells with an apicoplast (Fig. 2B, C). Quantitative analysis of progeny of parasites expressing the chimeric construct showed that only about 20% stained for the luminal marker (Fig. 2D). In contrast almost all parasites had V^ap^ as revealed by ATrx1 or FtsH1 markers, whether or not the vacuoles were positive for the chimeric protein. These findings corroborate a previous study in which the apicoplast was rapidly eliminated but V^ap^ retained following expression of a PI3P-binding protein [27]. Taken together, the above data supports the possibility of two trafficking pathways: one for luminal proteins and one for ApV proteins. Furthermore, the similar abundance of V^ap^ bearing ATrx1 and FtsH1 in cells with and without an apicoplast indicates that V^ap^ do not arise from apicoplast.

### Relationship of V^ap^ to the Golgi body

**Figure 3:**
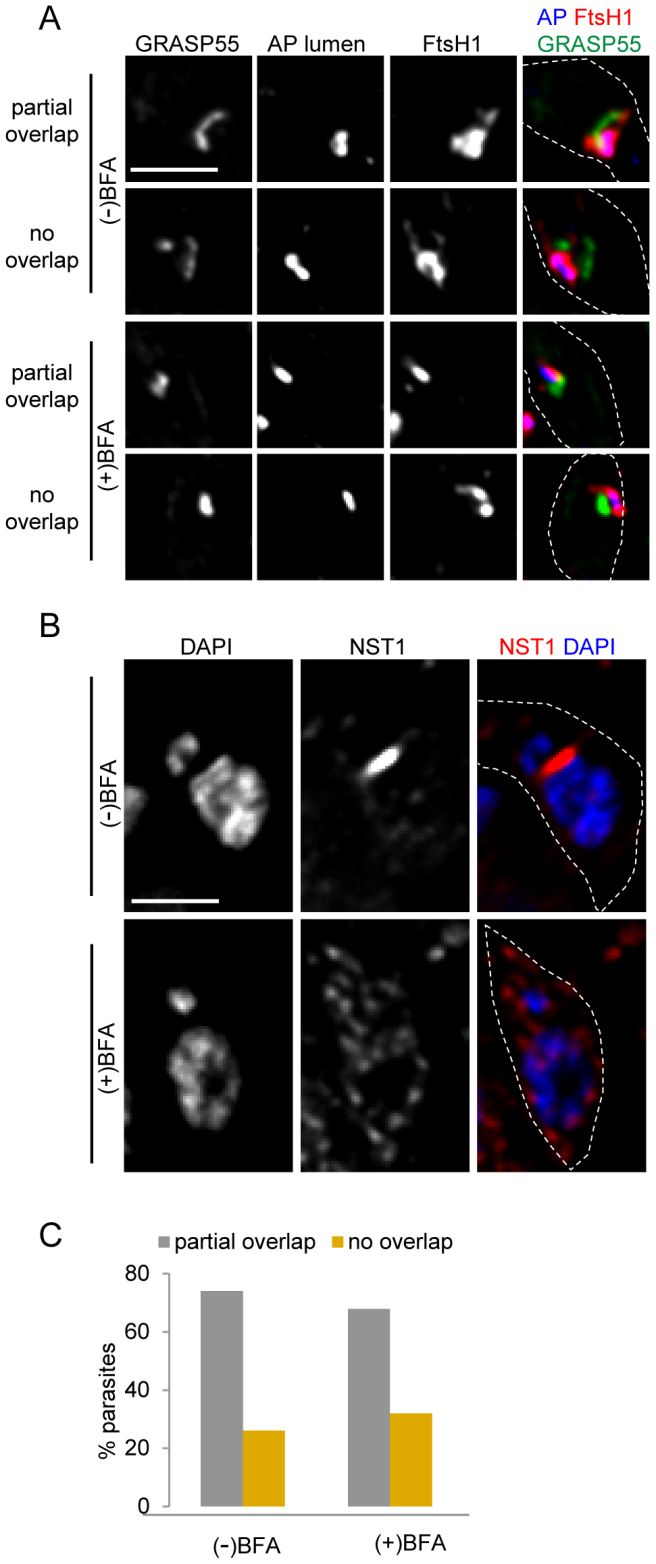
Overlap between FtsH1 and GRASP55 does not reflect FtsH1 protein in the Golgi body. A) Parasites were incubated with or without 1 µg/ml BFA for 1 hour at 37°C. *T. gondii* co-expressing the Golgi matrix marker GRASP55-YFP and FtsH1 internally tagged with V5 epitopes were stained with anti-V5 mAb followed by secondary antibody coupled to DyLight 649 and Texas Red streptavidin (which detects a naturally biotinylated protein in the apicoplast lumen, AP lumen). Bar, 2 µM. B) Parasites expressing the Golgi membrane protein NST1-HA (detected with anti-HA mAb coupled to Alexa 594) served as the control, demonstrating the effectiveness of BFA. Bar, 2 µM. C) Quantitative analysis of signal overlap between internally tagged FtsH1 and GRASP55 in the presence or absence of BFA. More than 50 parasites were analyzed for each condition (see Methods).

It is unknown whether ApV proteins follow a Golgi-independent pathway (similar to apicoplast luminal proteins) or whether they transit the Golgi, as seen for other proteins that exit the ER. If ApV proteins were to pass through the Golgi body, we might detect their presence in the organelle. While previous immunoelectron microscopy studies did not show evidence of localization of these proteins to the Golgi [22]–[24], those studies were limited by the number of relevant images analyzed. However, other studies were able to detect colocalization of the microneme proteins MIC2 and M2AP with the Golgi protein Rab51 as the former transit the Golgi body [31]. Here, we looked for colocalization of FtsH1 and the Golgi stacking protein, GRASP55 [32]. As seen in earlier work [33], the apicoplast and Golgi body are usually in close proximity. In some cells no overlap of the signal for the two proteins was visible. However, we often observed closely abutting or weak partial overlap of the two proteins and occasionally stronger signal overlap (Fig. 3A). To assess the functional significance of the overlap, we treated the intracellular parasites with BFA to disrupt the Golgi body. The Golgi membrane marker NST1 [25], [34], was distributed back to the ER after addition of BFA (Fig. 3B), demonstrating effective inhibition of ER-Golgi transport, while the Golgi stacking protein GRASP55, which is relatively resistant to BFA, maintained its position in the cell (Fig. 3A) as seen by others [32]. The pattern of overlap between FtsH1 and GRASP55 was maintained following BFA treatment (Fig. 3C), indicating that the observed overlap is likely not functional but rather reflects the closely juxtaposed positions of the organelles. Thus these experiments provided no indication that FtsH1 transiently inhabits the Golgi body.

**Figure 4:**
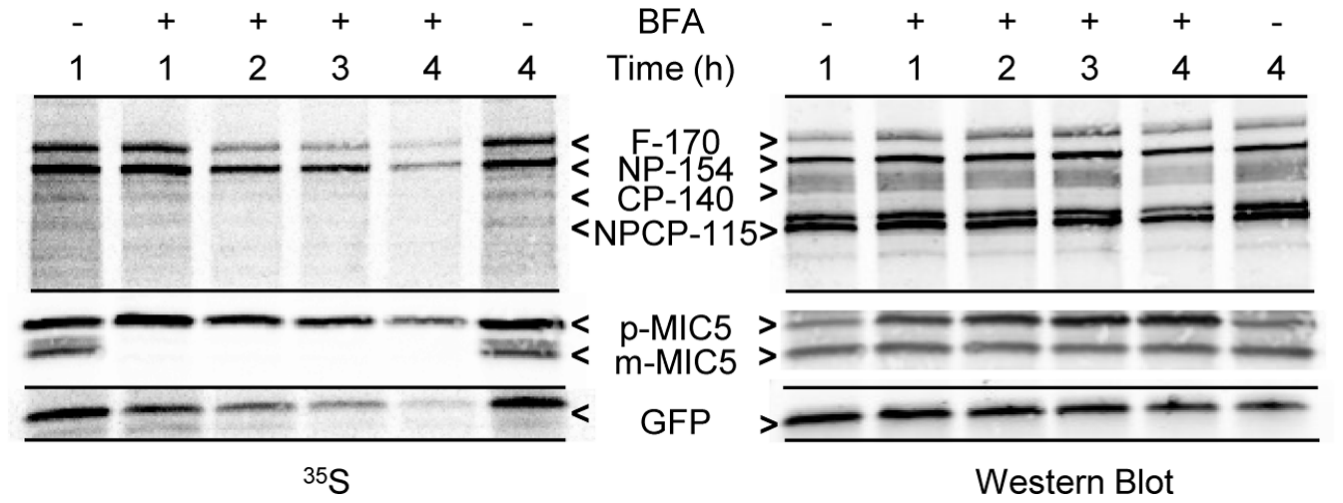
Protein synthesis during BFA treatment assessed by biosynthetic labeling of FtsH1, MIC5 and cytosolic GFP. Fibroblast monolayers infected with *T. gondii* expressing FtsH1 tagged internally with V5 epitopes and a cytosolic GFP (∼10^8^) were pre-incubated with or without BFA (1 µg/ml) for the indicated times prior to being labeled with ^35^S-methionine/cysteine for 30 minutes. Samples were immunoprecipitated with anti-V5 mAb, anti-GFP, and anti-MIC5 before being separated on 7.5% (FtsH1) or 8–16% (GFP and MIC5) SDS-PAGE gels and transferred to nitrocellulose. The left panel shows phosphorimaging, the right panel shows the same lanes detected by Western blot. The four major forms of FtsH1 are marked according to their apparent molecular mass on SDS-PAGE: full-length (F-170), N-terminally processed (NP-154), C-terminally processed (CP-140) or dual processed (NPCP-115). In a 30 min labeling, the first two forms predominate [21]. The precursor (p) and mature (m) forms of MIC5 [75] are marked.

ApV proteins could transit very rapidly through the Golgi body, thus escaping steady state detection. We therefore examined in detail the effect of BFA treatment on the presence of V^ap^. If V^ap^ represented ER to Golgi or Golgi to apicoplast intermediates, we would expect that a block of Golgi function would inhibit their formation. Although the Golgi marker NST1 relocalized to the ER within 15 min of the application of BFA (not shown), the drug might not affect the trafficking of previously formed Golgi to apicoplast intermediates. Thus, we aimed to incubate the parasites in drug as long as possible to allow pre-existing V^ap^ to arrive at their destination while still allowing protein synthesis to generate new V^ap^ cargo. Protein synthesis, assessed by ^35^S-methionine labeling of three proteins (FtsH1, the microneme protein MIC5, and cytosolic GFP), continued robustly for 1.5 hour after application of BFA, being very similar to the untreated control (Fig. 4). Subsequently, protein synthesis dropped precipitously in the BFA-treated parasites. Therefore we chose a 1.5 hour treatment with BFA for our IFA studies.

**Figure 5:**
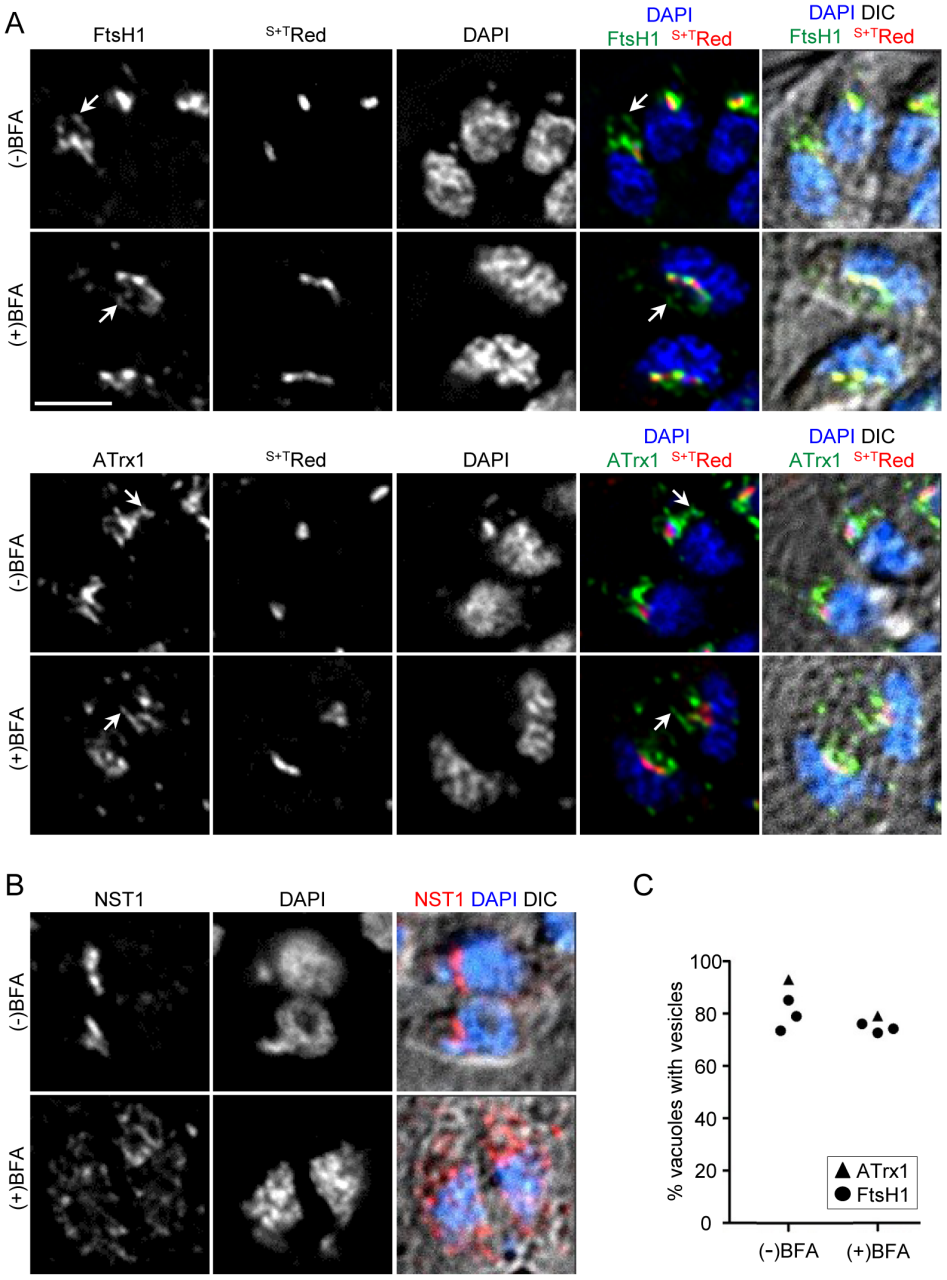
V^ap^ persist in the presence of the Golgi disruptor BFA. A) IFA analysis of *T. gondii* grown with or without 1 µg/ml BFA for 1.5 hours. The parasites expressed the luminal apicoplast marker ^S+T^Red along with FtsH1internally tagged with V5 epitopes or ATrx1-HA, which were detected with α-V5 followed by anti-mouse IgG (FITC) or anti-HA mAb directly coupled to FITC. Arrows indicate V^ap^-like structures in control and BFA-treated parasites. Bar, 2 µM. B) IFA of parasites expressing the Golgi membrane protein NST1-HA (detected with anti-HA mAb coupled to FITC), treated in parallel. Bar, 2 µM. C) Quantitation. The percentage of vacuoles with parasites bearing V^ap^ in the presence (+) or absence (−) of BFA is depicted. Three replicates are shown for FtsH1 (circle) and one for ATrx1 (triangle). More than 125 vacuoles were analyzed for each point. At the times chosen, the proportion of parasites with apicoplasts at stage 2 (elongated oval), stage 3 (elongated bar), and stage 4 (V-shaped bar) were: FtsH1 analysis: control, 82.7%; BFA, 72.2%; ATrx1 analysis: control, 81.6%; BFA, 74.5%.

Intracellular *T. gondii* expressing either epitope-tagged FtsH1 or ATrx1 were incubated with or without BFA and analyzed by IFA. We determined the proportion of vacuoles with parasites showing vesicles bearing the tagged proteins in BFA treated and control samples. Examples of such cells are shown in Fig. 5A and the quantitative analysis is shown in Fig. 5C. In these experiments, at the times chosen for analysis, a larger percentage of parasites were at stages 2–4 of the apicoplast division cycle [26] as compared to our previous studies [22], [23], accounting for the higher percentage of parasites bearing V^ap^. Parasites bearing FtsH1 and ATrx1 marked vesicles were observed in both control and BFA treated samples (Fig. 5A) even though the BFA treatment triggered re-distribution of NST1 from the Golgi body to the ER (Fig. 5B). After 1.5 hour in BFA, only a small reduction of the proportion of vacuoles showing V^ap^ was observed as compared to the untreated control (Fig. 5C). Since protein synthesis continues to be high within this period, newly synthesized ApV proteins must have accumulated in many parasites. However, these markers were not observed in a dispersed or perinuclear pattern as would be expected if they were retained in the ER upon Golgi disruption, as seen for other systems such as the trafficking of the plasma membrane protein VSV-G in CHO cells [35]. Thus V^ap^ persist and may continue to be formed in the absence of Golgi body function. As expected from the drop in protein synthesis, prolonged treatment with BFA (e.g., 4 hours) led to a dramatic decrease in V^ap^ (not shown).

**Figure 6:**
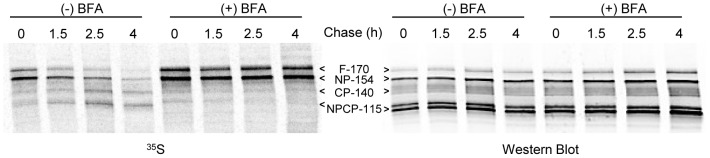
Effect of BFA on FtsH1 processing. Intracellular *T. gondii* expressing FtsH1 internally tagged withV5 epitopes were metabolically labeled for 30 min and then chased for various times in complete medium. BFA-treated samples included the drug throughout the pulse-chase. FtsH1 was then immunoprecipitated and subjected to SDS-PAGE followed by phosphorimaging (^35^S panel). The blot was subsequently probed with anti-V5 mAb (Western panel). The four major forms of FtsH1 are marked according to their apparent molecular mass on SDS-PAGE: full-length (F-170), N-terminally processed (NP-154), C-terminally processed (CP-140) or dual processed (NPCP-115).

FtsH1 is proteolytically processed at the N-terminus in the ER and then at the C-terminus around the time it reaches the apicoplast [21]. We therefore tested whether processing was occurring normally in the presence of BFA. If newly synthesized protein is retained in the ER, we would expect near-complete N-terminal processing as was seen with an ER-trapped FtsH1 mutant [21], but no C-terminal processing. Pulse-chase analysis (Fig. 6) showed that in presence of BFA, the N-terminus of FtsH1 was processed, but not to completion, suggesting that some molecules were inaccessible to the ER-associated processing machinery. Additionally, as discussed later, the C-terminus was not processed. Immunoblot analysis demonstrated that the total amount of FtsH1 with an intact C-terminus was not significantly increased in the 1.5 hour BFA treatment, most likely because most of the FtsH1 in the population was processed prior to the BFA treatment.

**Figure 7:**
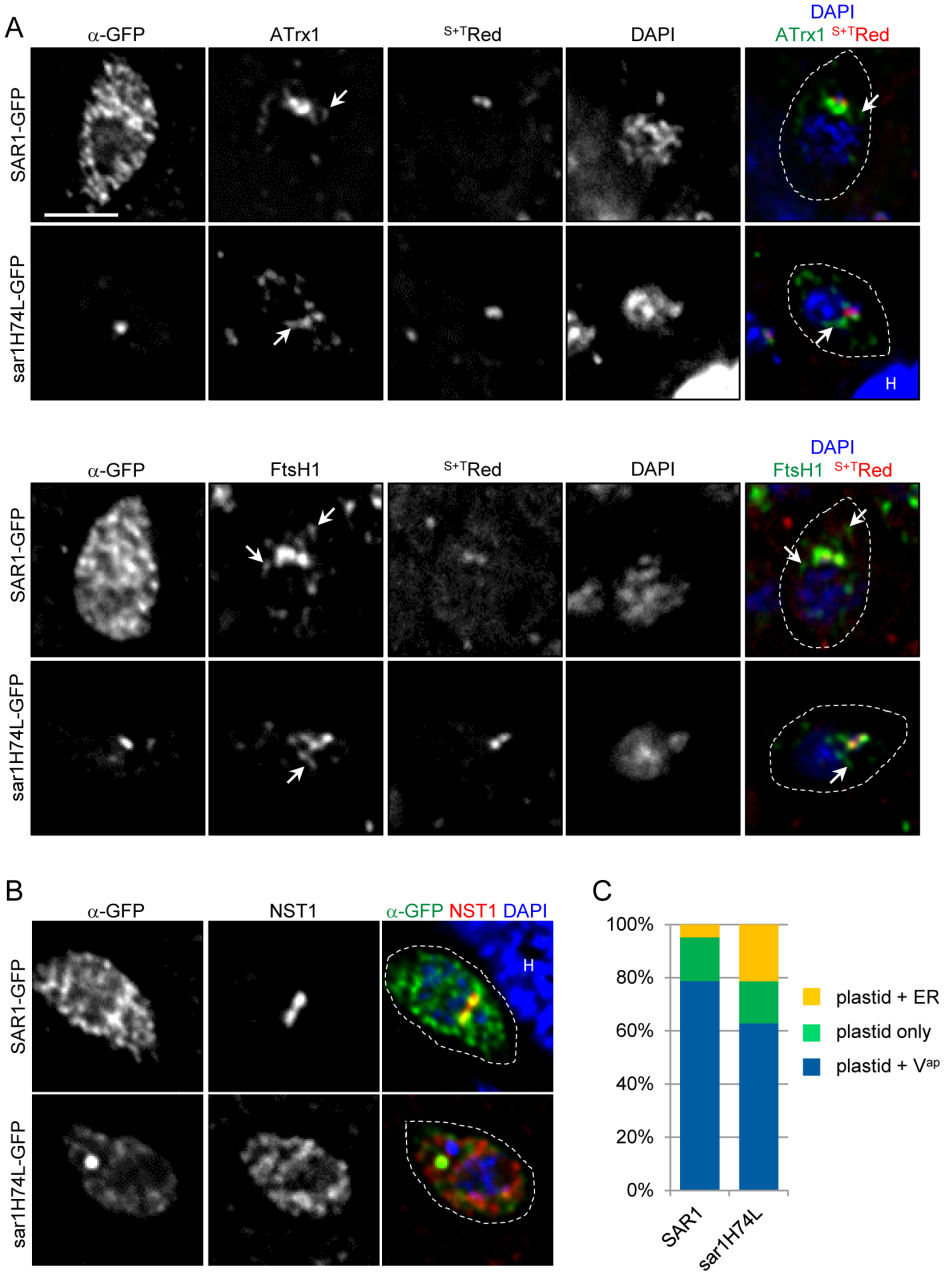
Expression of dominant negative *sar1* does not eliminate V^ap^. A) *T. gondii* expressing ^S+T^Red plus ATrx1-HA or FtsH1 internally tagged with V5 epitopes were transiently transfected with either wt SAR1-GFP or sar1(H74L)-GFP and analyzed by IFA for the localization of the two ApV protein. Epitope tagged proteins were detected by mAbs reactive with the epitope tags followed by secondary antibodies coupled to DyLight 649. The fluorescent proteins were detected by endogenous fluorescence. Arrows point to V^ap^-like structures. Bar, 2 µM. B) Overexpression of sar1(H74L) abrogates localization of NST1. SAR1 and sar1(H74L) constructs were transiently transfected into *T. gondii* expressing NST1-HA and the samples analyzed as above. Note the reticular staining of NST1 following expression of the dominant negative protein. C) V^ap^ are still present after induction of sar1(H74L) in stable transfectants. As described in Methods, parasites were stably transfected with constructs bearing sar1(H74L)-YFP or the wt SAR1-YFP that was separated from a promoter by RFP flanked by *loxP* sequences. Addition of rapamycin led to excision of the RFP sequence that separated and expression of the test proteins. After 11 hours YFP^+^ parasites were scored for the presence or absence of V^ap^ using mAb 11G8 which detects ATrx1 (followed by secondary antibody coupled to DyLight 350).

To further probe the relationship of V^ap^ to the Golgi body, we complemented the BFA studies with a genetic method to disrupt Golgi function. The small GTPase Sar1 plays an essential role in vesicular trafficking between the ER and the Golgi, and a dominant negative form of Sar1 has been shown to disrupt ER to Golgi trafficking in other systems [36]–[39]. Cycling between cytosolic and membrane-associated pools, Sar1 accumulates at ER exit sites, where insertion of its N-terminal α-helix initiates vesicle budding [40], [41]. In mammalian cells, Sar1 is localized uniformly across the ER membrane aside from some accumulation at ER exit sites [42], [43]. In *P. falciparum*, PfSar1p tagged with GFP at either the N-terminus or the C-terminus is associated with the ER throughout the erythrocytic cycle [44]. Sar1 is conserved in *T. gondii*, including the residue typically mutated to create a dominant negative form (histidine 74 in the *T. gondii* sequence) [45]. We generated *T. gondii* sar1(H74L) and fused the coding sequence, as well as wild type (wt) SAR1, to GFP. Constructs were transiently transfected into parasites expressing various markers for the Golgi body and apicoplast. Parasites expressing sar1(H74L)-GFP showed a delay in replication; therefore we examined the location of the above proteins at 11 hours post-transfection when all vacuoles contained one parasite. SAR1-GFP partially co-localized with the ER-resident protein BiP [46] and with GRASP55 (Fig. S4), as well as showing a diffuse staining which may represent the cytosolic pool. In contrast sar1(H74L)-GFP is locked at the Golgi body, colocalizing with GRASP55 (Fig. S4). This localization is different from what has been observed in mammalian cells where the corresponding mutant is localized at clustered ER exit sites [47]. The overexpression of SAR1-GFP did not alter the trafficking of NST1 to the Golgi body suggesting that the secretory pathway was not disrupted by overexpression of the wt protein (Fig. 7B). In contrast, expression of sar1(H74L)-GFP disrupted NST1 targeting, such that the protein was observed in a reticular structure characteristic of the ER (Fig. 7B). Turning to the trafficking of ApV proteins, overexpression of either SAR1-GFP or sar1(H74L)-GFP led to the same effect: V^ap^ bearing ATrx1 and FtsH1 continued to be observed (Fig. 7A)

### Conditional expression of dominant negative sar1 by promoter juxtaposition

We were unable to obtain stable transfectants expressing sar1(H74L)-GFP upon selection and so developed an approach to conditionally overexpress the mutant or wt protein making use of the di-CRE system [48] that was recently applied to *T. gondii* [49]. Constructs were prepared in which wt or mutant SAR1 fused toYFP was separated from the *TUBA* promoter by a segment of DNA encoding a red fluorescent protein flanked by *loxP* sites. After stable transfection into parasites expressing two inactive fragments of the Cre recombinase (DiCre) [49], assembly of functional CRE was initiated by the addition of rapamycin (Fig. S5A). This treatment should lead to the excision of the DNA between the *loxP* sites and hence juxtaposition of the promoter and the *SAR1/sar1-YFP* fusion genes. The fusion proteins migrated according to the expected molecular mass on SDS-PAGE (Fig. S5B), although the H74L mutant was expressed to lower levels. Localization of the wt and mutant fusion proteins corresponded to those seen in transient transfectants (Fig. S5D). The percentage of parasites expressing the SAR1/sar1 fusion proteins in clonal lines increased gradually over 24 hours, when 70–80% showed visible expression (not shown). We observed cellular abnormalities such as lack of elongation of the inner membrane complex and aberrant micronemes (as revealed by Mic10) within 13 hours of rapamycin addition to induce sar1(H64L); small cells began to appear by 16 hours and by 24 hours the majority of cells were shrunken (not shown). These sar1(H64L)^+^parasites were lost upon cultivation (Fig. S4C). Hence we aimed to use the earliest times possible for analysis to avoid secondary affects.

At 8 hours after rapamycin treatment, in those cells where the sar1(H74L) fusion protein was detected Golgi function was not yet compromised, while at 11 hours disruption of NST1 localization to the Golgi body was evident (Fig. S5D). We therefore examined parasites for V^ap^ 11 hours after rapamycin induction. Approximately 75% of those parasites expressing the wt SAR1 protein showed V^ap^, while 60% of parasites expressing sar1(H74L) did (Fig. 7C and Fig. S5E). This modest decrease in V^ap^ in the parasites expressing the dominant negative sar1 was paralleled by an increase in parasites showing ER localization of ATrx1. However, it is unclear whether ER retention of ATrx1 in this population is a primary effect of Golgi disruption. Because of the gradual onset of detectable expression and the pleiotropic effect of Sar1 disruption, biochemical studies were not pursued.

## Discussion

The work described here adds to the understanding of the trafficking of apicoplast proteins in several ways. First, it demonstrates that the luminal marker protein travels to the apicoplast by routes largely independent of the pathway generating V^ap^. It shows that the presence, and possibly the formation of V^ap^, does not require an intact Golgi body. Finally it supports work suggesting that V^ap^ are not derived from the apicoplast [27] by revealing their continued presence several cell generations after plastid loss. The persistence of V^ap^ in such parasites indicates that a retrograde pathway is not required for their formation.

### Relationship of V^ap^ to the Golgi body

Precedents exist for protein trafficking from the ER to organelles but bypassing the Golgi body, such as pathways generating the bounding membranes of lipid droplets [50], [51] and peroxisomes. However, in *Euglena* (which arose from a separate evolutionary lineage from the apicoplast) and in *Gonyaulax* (which is thought to be in the same lineage as the apicoplast), proteins clearly transit the Golgi body to reach the secondary plastid [52]–[54]. While a recent study suggested that a *Plasmodium* thioredoxin peroxidase reaches the apicoplast via the Golgi body [55], trafficking of most luminal proteins to the apicoplast appears to be Golgi-independent [11], [12]. We therefore assessed potential Golgi involvement in V^ap^ and membrane protein trafficking. Although fluorescence microscopy images of *T. gondii* apicoplast proteins and Golgi stacking protein GRASP55 often had partially overlapping signals, we showed that such overlap is maintained even when the Golgi body membranes and contents were relocalized to the ER by treatment with BFA. Thus, this apparent colocalization simply reflects the close juxtaposition of the organelles and does not imply intersection of V^ap^ proteins with the Golgi during trafficking. To more directly test the role of the Golgi body, we used both the chemical inhibitor BFA and the genetic inhibitor sar1(H74L) to disrupt Golgi function. In both cases, we observed V^ap^ in the treated cells, indicating that they either persist or continue to be formed when Golgi body function is abrogated. Longer term Golgi disruption does lead to loss of V^ap^ (4 hours of BFA treatment, from which parasites cannot recover), although by that time numerous cellular organelles and activities, including protein synthesis, have been compromised.

While the microscopic studies do not support a role for the Golgi body in ApV protein trafficking, changes in FtsH1 processing following BFA treatment complicate the issue. Processing of the N-terminus, which occurs in the ER, is reduced (especially as compared to an ER-trapped mutant FtsH1 [21]) and C-terminal processing, thought to occur at the apicoplast, is blocked. Regarding the former, if the formation of V^ap^ does not require the Golgi body, FtsH1 could continue to be packaged into V^ap^ in the presence of BFA, thereby escaping the ER-associated processing activities. Alternatively ER-associated processing activity could be swamped by the presence of fortuitous substrates made available by BFA inhibition of secretory trafficking to the Golgi body. The lack of C-terminal processing suggests that no FtsH1 reaches the apicoplast, but other explanations cannot be ruled out. For example, the ACP transit peptide is not cleaved upon BFA treatment even though the protein co-localizes with the apicoplast [11] (although in *P. falciparum* transit peptide cleavage continued following similar treatment [12]). The collapse of the Golgi body could enable proteins bearing (fortuitous) weak apicoplast targeting sequences to localize to the plastid, where they could inhibit or overwhelm processing enzymes. Alternatively, BFA could inhibit a later step in FtsH1 trafficking. For example, in diatoms (which also possess secondary plastids) BFA treatment results in the accumulation of plastid proteins in a structure at the plastid boundary as visualized by fluorescence microscopy [56].

### Function of V^ap^

To test whether V^ap^ bear apicoplast luminal proteins in addition to ApV proteins, we examined the potential colocalization of marker proteins driven by apicoplast gene promoters, using conditions facilitating detection of the luminal marker ^S+T^Red-V5. Most V^ap^ lacked signal from the luminal marker altogether. In the occasional cases where we could detect colocalization of the luminal marker and ApV proteins in structures microscopically distinct from the apicoplast, the relative signal of the luminal marker compared to the apicoplast was much lower (10%, just above background) than that of the canonical ApV protein ATrx1 (50%). In parasites lacking a plastid, the ApV proteins accumulated in structures near the site where the apicoplast is usually found, but the luminal marker was not detected in those structures. Thus trafficking of the luminal marker protein, and most likely other luminal proteins, to the apicoplast appears to be largely independent of V^ap^.

The functional role of V^ap^ remains unclear. While they do not appear to contain luminal proteins, two findings link them with trafficking to the apicoplast. These are the visualization of apparent V^ap^ fusions with the apicoplast by immunoelectron microscopy [23] and the demonstration that the same determinants required for routing the membrane protein APT1 to the apicoplast are also required for its recruitment to V^ap^ [25]. Nonetheless, it remains possible that the primary function of V^ap^ is not protein trafficking. The apicoplast sports four membranes, all of which need lipid building blocks for growth and replication. Although the lipid composition of the apicoplast membranes is unknown, filipin disrupts the outer membrane(s) [57] indicating the presence of high levels of sterols, which are acquired from the host cell [58]. The apicoplast itself synthesizes fatty acids; these are further elongated and incorporated into phospholipids in the ER [59], and then by analogy with other plastids [60], re-imported for use in plastid membranes. Plastid to ER transport occurs via acyl-coAs rather than vesicular trafficking [60], [61]. In contrast ER to plastid lipid trafficking is proposed to occur via membrane interactions [62]. V^ap^ may provide such a mechanism for bulk transport of sterols and elongated lipids from the ER to the apicoplast. Finally, the data are compatible with V^ap^ being relatively persistent structures, only some of which may fuse with the apicoplast. In that case, a distinct Golgi-mediated pathway for routing membrane proteins might also be postulated.

### Application of the DiCre system for expression of a toxic sar1 mutant protein

In transient transfections we observed that expression of sar1(H74L) slowed parasite replication such that stable lines expressing the protein could not be generated, necessitating a conditional approach. Several molecular genetic approaches have been developed that allow conditional gene expression to test gene function in *T. gondii*. These include regulated induction [63] or repression [64] of transcription by tetracycline and regulated degradation of proteins fused to destabilization domains by small molecules such as Shld [65]. More recently, the DiCre system was instituted in *T. gondii*, allowing the excision of gene sequences to examine gene function. The approach we used here is a modification of the latter, in which gene expression is induced by excising transcription termination sequences [66]. Such an approach is particularly useful when studying potentially toxic mutant proteins [67], especially in cases where regulated degradation cannot be fully achieved. In our case, because excision induced by rapamycin is efficient in *T. gondii*, the majority of treated parasites expressed the tagged sar1 (or SAR1) allele and it was possible to examine the phenotype in the pool of cells. This adaptation may be useful for studying other proteins for which dominant negative alleles may be created.

## Materials and Methods

### Cell culture, molecular cloning and transfection

*T. gondii* were grown in primary human foreskin fibroblasts provided by Dr. William Carter at the Fred Hutchinson Cancer Research Center, who obtained them as coded samples from Swedish Medical Center. Our IRB board (Western IRB) advised us that because these are coded biological samples, their use does not constitute human subjects research. This is in agreement with the U.S. Department of Health and Human Services (http://www.hhs.gov/ohrp/sachrp/20110124attachmentatosecletter.html. Among the strains used were RH and its corresponding *HXGPRT* deletion strain [68], plus derivatives expressing apicoplast proteins C-terminally tagged with four HA epitopes including ATrx1 (ToxoDB v.7.2 TGME49_312110) [23], APT1 (TGME49_261070) [24], and FtsH1 (TGME49_259260), which was additionally internally tagged with two V5 epitopes at residue 233 (FtsH1-V5^233^-HA) [21]. Some parasites also expressed *Heteractis crispa* red fluorescent protein HcRed bearing the ACP (TGME49_264080) signal plus transit sequences and tagged with V5 epitope (^S+T^Red-V5). The two V5 tags (see [21] for sequence) were inserted into *Mun*I and *Nde*I sites that had been added by site directed mutagenesis using 5′ CCCGAGAAGGCCAACCAATTGATACATATGTGACTGCAGCCCACACAG 3′ and 5′ CTGTGTGGGCTGCAGTCACATATGTATCAATTGGTTGGCCTTCTCGGG 3′. Expression of each of these proteins was driven by its cognate promoter. Tic22 (TGME49_286050) [19] was amplified from *T. gondii* RH strain cDNA using primers CTCAGATCTAAAATGGGCTTCATCGCTCTCCG and GTGCCTAGGTGCTTGTCCTTGATCGTCGG, and cloned into a pGem shuttle vector. The relevant region was excised with *Bgl*II and *Xba*I and cloned into the plasmid pHX apt1:APT1-4HA that had been digested with *Bgl*II and *Avr*II to remove the APT1 coding sequence. The product yielded Tic22 C-terminally tagged with HA, with expression is driven by the *APT1* promoter, which has similar kinetics. Der1ap (Genbank FJ976520) [19], was similarly amplified using primers GTGCCATGGAAAGAGGGGATTTTTTCTC and CACTCTAGAGCGTTTCCAACGGCGTCCTCG, cloned into a pGem shuttle vector and then excised with *Nco*I and *Avr*II. The appropriate fragment was cloned into pHX ATrx1:ATrx1-4HA that had been digested with the same enzymes to remove the ATrx1 coding sequence. The resulting construct encoded Der1ap C-terminally tagged with HA, with its expression is driven by the *ATrx1* promoter. GRASP55-HcRed was generated by replacing the YFP tag in pCAT GRASP55-YFP with the HcRed tag from pCAT ACP-HcRed, using *Avr*II and *Pst*I. Other markers include ^S+T^Red (untagged), the Golgi membrane protein NST1 (TGME49_267380) C-terminally tagged with HA [25], the Golgi stacking protein GRASP55 (AF110267) fused to YFP [32], [69] or HcRed, and GFP used as a cytosolic marker. Expression of ^S+T^Red and NST1 were driven by the *DHFR* promoter and expression of GRASP55-YFP, GRASP55-HcRed, and GFP was driven by the *TubA* promoter. Plasmids were transfected into *T. gondii* by electroporation and stable transfectants selected either with chloramphenicol or mycophenolic acid plus xanthine as previously described [24], and clonal lines were isolated. Brefeldin A (Calbiochem) was used at a final concentration of 1 µg/ml, previously shown to be sufficient to block trafficking of the microneme protein MIC5.

The *T. gondii* small GTPase Sar1 (TGME49_215060) was amplified from oligo-dT primed cDNA using 5′ ATCGAGATCTAAAATGTTCGTCTTCAACTGGTTCTG 3′ and 5′ ATCGCCTAGGGTTGAGGAACTGAGACAACCAAC 3′ and cloned into pCAT GFP [70] cleaved with *Bgl*II and *Avr*II. Its expression is driven by the *TubA* promoter (*TubA* mRNA abundance and cell cycle kinetics are similar to that of *SAR1*). The H74L mutant was generated by site-directed mutagenesis using the pCAT Sar1-GFP plasmid as a template and primers: 5′ TTCGATCTTGGGGGACTTGAAACAGCC 3′ and 5′ GGCTGTTTCAAGTCCCCCAAGATCGAA 3′. An apicoplast “poison” construct was kindly provided by Dr. Cynthia He. This plasmid encodes an apicoplast-targeted fusion protein that also contains sequences from the rhoptry protein Rhop1 (FNR-YFP-ROP1), and is identical to that previously used by He et al. [71] except that the apicoplast targeting sequence was derived from ferredoxin reductase rather than ACP. These plasmids were employed in transient transfections.

The plasmid p*loxP-KillerRed-loxP-YFP*, in which expression of Killer Red was driven by the *Tub8* promoter, was a gift from Drs. Markus Meissner and Nicole Andenmatten [49]. It bears the selectable marker *HXGPRT*. The plasmid and was modified by inserting an *Xba*I restriction site upstream of YFP using the oligonucleotides lox-Xba-YFP 5′ CATTATACGAAGTTATAAATCTAGAATGGTGAGTAAGGGCGAGGAG 3′ and 5′ CTCCTCGCCCTTACTCACCATTCTAGATTTATAACTTCGTATAATG 3′. A segment of genomic DNA downstream of the *GRA3* locus was amplified from genomic DNA using primers Gra3-tub8 f 5′ ATTGGGTACCGGGCCCTACGGTCTCCTAGCTCCTTTG 3′ and r 5′ CGTCGAGGGGGGGCCGTGAGAATCGTAGGTGCAGGTG 3′, and inserted into the Apa1 site the plasmid by Gibson cloning. The coding regions of *SAR1* and sar1(H74L) were amplified from the above pCAT plasmids using oligonucleotides P-lox-YFP-SAR1 f 5′ CATTATACGAAGTTATAAATCTAGAATGTTCGTCTTCAACTGGTTCTGG 3′ and r 5′ GCCCTTGCTCACCATTCTAGAGTTGAGAAACTGAGACAACCAACG 3′ and inserted into *Xba*1-digested plasmid using Gibson cloning. The SAR1 coding regions in the plasmids were verified by sequencing.

For transfections, 50 µg of each plasmid (pGra3-*loxP*-Killer red YFP vector and SAR1-YFP and sar1 (H74L)-YFP derivatives) were digested with *Pae*I and transfected into RH Δ*KU80* Δ*HXGPRT* DiCre *T. gondii* and selected with mycophenolic acid and xanthine. Clonal cell lines were isolated by limiting dilution. Excision of the sequence separating the promoter from the SAR1/sar1 CDS was induced by 50 nM rapamycin in 0.1% DMSO. ATrx1 localization was categorized as plastid, plastid+ER or plastid+V^ap^ based on the localization within the majority of parasites within a vacuole.

### Pulse labeling, immunoprecipitation and immunoblot analysis

Fibroblast monolayers bearing *T. gondii* (approximately 10^8^) were rinsed twice in medium lacking methionine and cysteine, with or without BFA. Intracellular parasites were labeled for 30 min with 100 µCi/ml [^35^S] trans label (methionine and cysteine, MP Biomedicals, Irvine, CA and Perkin Elmer) as described, in the presence or absence of BFA [11]. Subsequently the labeling medium was replaced with complete medium and the incubation continued for the indicated times. The fibroblast layer was then scraped from the flask and cells were pelleted by centrifugation at 2300× g for 2 min. Pellets were lysed in 0.5 ml lysis buffer (150 mM NaCl, 50 mM TrisHCl pH 7.5, 2 mM EDTA, 1% NP-40, 0.25% deoxycholate, 1.7 µg/ml aprotinin, 5 µg/ml leupeptin, 1 µM pepstatin, 0.1 mM PMSF). FtsH1-V5^233^-HA and ^S+T^Red-V5 were immunoprecipitated using mouse anti-V5 mAb (Invitrogen), and ATrx1-HA was immunoprecipitated with anti-HA mAb. GFP and MIC5 were immunoprecipitated with anti-GFP B2 (Santa Cruz Biotechnology), and rabbit anti-MIC5 (gift of Dr. Vern Carruthers) respectively. Immune complexes were collected with Protein G coupled to magnetic beads (Invitrogen). The washed immune complexes were separated by SDS-PAGE, transferred to nitrocellulose membranes. Radiolabeled proteins were detected by phosphorimaging using a Storm 860 (Molecular Dynamics).

Immunoprecipitated samples or total cell lysates (approximately 10^7^ parasites) were used for immunoblot analyses. After blocking in Odyssey block (LI-COR Biosciences), blots were probed with mouse anti-V5 mAb at 0.5 µg/ml (Invitrogen), rabbit anti-GFP at 0.2 µg/ml (Invitrogen) and rabbit anti-MIC5 at a 1∶10,000 dilution. This was followed by goat anti-mouse Ig coupled to IRDye 800 (1∶10,000, LI-COR) or goat anti-rabbit Ig coupled to IRDye 680 (1∶10,000, LI-COR). Membranes were scanned using an Odyssey infrared imaging system (LI-COR) and analyzed using the system software.

### Microscopy

For IFAs, parasites were grown overnight unless otherwise noted within fibroblasts monolayers on coverslips. IFAs were performed as described [24]. V5 tags were detected using mouse anti-V5 mAb IgG2a at 1 µg/ml (Invitrogen), followed by goat anti-mouse IgG2a FITC, goat anti-mouse IgG2a Texas Red, (Southern Biology), or goat anti-mouse IgG DyLight 649 (Thermo Scientific), all at 2 µg/ml. FtsH1-V5^233^-HA was always detected using anti-V5 mAb. ATrx1 and APT1 were detected by virtue of the HA tags, using FITC-coupled rat anti-HA mAb 3F10, 3 µg/ml (Roche), or anti-HA mAb16B12 (Covance) followed by goat anti-mouse IgG DyLight 649. ATrx1 was also detected by mAb 11G8 [72], [73], a kind gift from Peter Bradley) followed by goat anti-mouse IgG Dylight 350 (Thermo Scientific). Markers for the apicoplast lumen included the naturally biotinylated apicoplast luminal protein acetyl coA carboxylase revealed by Texas Red or Alexa 680 coupled streptavidin (Invitrogen, 1 µg/ml) [74], and ^S+T^Red or ^S+T^Red-V5 [70]. Rabbit anti-MIC5 was used at a 1∶500 dilution, rabbit anti-*Trypanosoma brucei* BiP (which cross-reacts with *T.gondii* BiP, gift of Dr. Jay Bangs) at a 1∶200 dilution [46] and 4,6-diamidino-2-phenylindole (DAPI) was used to stain the DNA. A Deltavision RT deconvolution microscope with an Olympus UPlan/Apo 100× 1.35 NA objective was used to view the slides. Images were deconvolved using softWoRx (version 3.5.1) using standard parameters and a conservative ratio algorithm. Single deconvolved planes are shown except as described below.

### Image quantitation

To quantify relative fluorescence in vesicles or adjacent regions *versus* in the apicoplast, the softWoRx “quick projection sum” tool was used to generate a 2-D image showing the summed fluorescence at each pixel from each plane of the 3-D image; the resulting image was then converted to a TIFF file for further analysis. Using Metamorph software, three ovals of 30 pixels each were placed in each subcellular region analyzed: the apicoplast region defined by ^S+T^Red-V5 (Cy5 channel) and ATrx1-HA (FITC channel), vesicle regions (defined by ATrx1 punctate signal), and adjacent regions (areas next to ATrx1 vesicle regions). Average fluorescence per oval in both FITC and Cy5 channels was calculated and summed for each region (90 pixels) in each parasite. Ten parasites from separate vacuoles were analyzed. Background was calculated by placing all nine ovals on images of the apical end of co-cultured RH parasites (which do not express either tagged protein) and the corresponding average fluorescence per 90 pixels was calculated for each channel, averaged over four parasites.

To assess potential localization of ApV proteins in the Golgi body, images from treated and non-treated samples were scaled equally and colocalization of organellar markers was examined in all image planes. Individual parasites were scored as having abutting/partial overlap if regions of two or more pixels wide in each channel overlapped in two or more focused image planes.

## Supporting Information

Figure S1 **Cell cycle regulation of transcription of genes in this study.** Quantitation of expression of the relevant genes following post-thymidine block release of *T. gondii* RH^TK+^ parasites was obtained from ToxoDB, based on microarray data from [28]. The time period covers approximately 1.66 cell cycles, with internal daughter cells peaking at 4 and 12 hours. These data show that the promoters for apicoplast reporters utilized in this study have very similar temporal kinetics.
(TIF)

Figure S2 **Similar half-life of ^S+T^Red-V5 and ATrx1.** Pulse-chase analysis was carried out as in Fig. 6, with ^S+T^Red-V5 and ATrx1-HA co-expressed in the same parasite line. The molecules were sequentially immunoprecipitated with mAbs directed against the epitope tags and ^35^S-methionine labeled proteins detected by phosphorimaging. For each antibody, the lanes shown are from the same scan of the gel. Three main bands are seen for ATrx1-HA, with the 90 kDa protein being a precursor (p) to intermediate (i) and mature 65 kDa protein (m) [23]. The subcellular location where processing occurs is not known. The cleavage of the precursor (p) ^S+T^Red-V5 to mature form (m, 35 kDa) occurs within the apicoplast.
(TIF)

Figure S3 **Tic22 and Der1ap inhabit V^ap^.** Clonal lines expressing the apicoplast luminal marker ^S+T^Red and either Tic22-HA or Der1-HA *T. gondii* within fibroblasts were processed for IFA and stained for ATrx1 (using mAb 11G8 followed by anti-mouse IgG coupled to DyLight 649) and for Tic22-HA or Der1-HA (using rat anti-HA mAb coupled to FITC). Slides were co-stained with DAPI. The apicoplast luminal marker ^S+T^Red was detected by endogenous fluorescence. Dotted lines indicate outline of parasites within vacuole. A) Localization of Tic22-HA. Red, ATrx1; green, Tic22-HA. Panel A′ shows images with enhanced scaling to reveal V^ap^ and a merge image showing DIC (grey), DAPI (blue) Tic22 (green) ^S+T^Red (orange) and ATrx1 (maroon). Arrows indicate V^ap^ containing both Tic22 and ATrx1. B) Localization of Der1ap-HA. Red, ATrx1; green, Der1-HA. Panel B′ shows images with enhanced scaling to reveal V^ap^ and a merge image showing DIC (grey), DAPI (blue) Der1 (green) ^S+T^Red (orange) and ATrx1 (maroon). Arrows indicate V^ap^ containing both Der1 and ATrx1.
(TIF)

Figure S4 **Localization of SAR1-GFP and sar1(H74L)-GFP in** ***T. gondii*** **.** The constructs were transiently transfected into *T. gondii* expressing GRASP55-HcRed. After 11 hours, the samples were fixed and subjected to IFA, comparing the localization of SAR1 and sar1(H74L) (endogenous green fluorescence) to GRASP55 and BiP, an ER marker protein detected anti-*T. brucei* BiP followed by anti-rabbit IgG coupled to Alexa 680. Arrow marks colocalization of sar1(H74L)-GFP and GRASP55. This particular cell has duplicated its Golgi body. “H” marks a host cell nucleus. Bar, 2 µM.
(TIF)

Figure S5 **Conditional expression of sar1(H74L)-YFP in** ***T. gondii*** **.** A) Map of expression locus before (top) and after (bottom) excision of the *loxP*-flanked red fluorescent protein coding sequence which separates the promoter from the SAR1-YFP fusion proteins. Translated segments are indicated by dark fill with a line above.B) Western blot of protein from the parental RH parasites, and 24 hour rapamycin induced sar1(H74L)-YFP and SAR1-YFP parasites probed with anti-GFP. M, markers. The fusion proteins migrated at the expected size (49 kDa). C) Parasites expressing sar1(H74L) are lost upon cultivation. After rapamycin mediated induction of expression (via excision of the RFP gene), the percentage of vacuoles in each population with parasites expressing YFP-tagged SAR1 or sar1(H74L) was monitored over time. All parasites in a given vacuole showed the same expression phenotype. Before excision (day 0) both parasite lines showed red fluorescence only. For an intermediate period many parasites expressed both yellow fluorescent protein and previously transcribed and translated red fluorescent protein. Those parasites in which expression of SAR1-YFP was induced continue to grow and became YFP^+^/RFP^−^, whereas those expressing sar1(H74L)-YFP did not survive and were outgrown by the minority population that had not excised the RFP coding sequence (n>200 vacuoles for each time point). D) The conditionally expressed mutant sar1(H74L) disrupts the Golgi body. The SAR1/sar1 clonal parasite lines were transiently transfected with NST1-HA and after 15 hours rapamycin was added. Parasites were analyzed 11 hours later and representative examples are shown. Blind analysis indicated that NST1-HA was localized to the Golgi body in 95% of parasites expressing SAR1-YFP, but was redistributed to the ER in 81% of those parasites expressing sar1(H74L). E) V^ap^ persist in parasites expressing dominant negative sar1(H74L). Expression of wt or mutant SAR1 was induced by the addition of rapamycin and after 11 hours parasites were analyzed. Representative images are shown, detecting the ApV protein ATrx1 with mAb 11G8 and the apicoplast lumen with streptavidin as described in Methods.
(TIF)

## References

[1] Tenter AM, Heckeroth AR, Weiss LM (2000) *Toxoplasma gondii*: from animals to humans. Int J Parasitol 30: 1217–1258. 11113252 10.1016/s0020-7519(00)00124-7 PMC3109627

[2] Dubey JP, Jones JL (2008) *Toxoplasma gondii* infection in humans and animals in the United States. Int J Parasitol 38: 1257–1278. 18508057 10.1016/j.ijpara.2008.03.007

[3] Seeber F (2002) Biogenesis of iron-sulphur clusters in amitochondriate and apicomplexan protists. Int J Parasitol 32: 1207. 12204220 10.1016/s0020-7519(02)00022-x

[4] Seeber F, Soldati-Favre D (2010) Metabolic pathways in the apicoplast of apicomplexa. Int Rev Cell Mol Biol 281: 161–228. 20460186 10.1016/S1937-6448(10)81005-6

[5] Waller RF, Keeling PJ, Donald RGK, Striepen B, Handman E, et al (1998) Nuclear-encoded proteins target to the plastid in *Toxoplasma gondii* and *Plasmodium falciparum* . Proc Natl Acad Sci USA 95: 12352–12357. 9770490 10.1073/pnas.95.21.12352 PMC22835

[6] Jomaa H, Wiesner J, Sanderbrand S, Altincicek B, Weidemeyer C, et al (1999) Inhibitors of the nonmevalonate pathway of isoprenoid biosynthesis as antimalerial drugs. Science 285: 1573–1576. 10477522 10.1126/science.285.5433.1573

[7] Ralph SA, van Dooren GG, Waller RF, Crawford MJ, Fraunholz MJ, et al (2004) Tropical infectious diseases: metabolic maps and functions of the *Plasmodium falciparum* apicoplast. Nat Rev Microbiol 2: 203–216. 15083156 10.1038/nrmicro843

[8] Fichera ME, Roos DS (1997) A plastid organelle as a drug target in apicomplexan parasites. Nature 390: 407–409. 9389481 10.1038/37132

[9] Budimulja AS, Syafruddin, Tapchaisri P, Wilairat P, Marzuki S (1997) The sensitivity of *Plasmodium* protein synthesis to prokaryotic ribosomal inhibitors. Mol Biochem Parasitol 84: 137–141. 9041529 10.1016/s0166-6851(96)02781-8

[10] McConkey GA, Rogers MJ, McCutchan TF (1997) Inhibition of *Plasmodium falciparum* protein synthesis. Targeting the plastid-like organelle with thiostrepton. J Biol Chem 272: 2046–2049. 8999899 10.1074/jbc.272.4.2046

[11] DeRocher A, Gilbert B, Feagin JE, Parsons M (2005) Dissection of brefeldin A-sensitive and -insensitive steps in apicoplast protein targeting. J Cell Sci 118: 565–574. 15657083 10.1242/jcs.01627

[12] Tonkin CJ, Struck NS, Mullin KA, Stimmler LM, McFadden GI (2006) Evidence for Golgi-independent transport from the early secretory pathway to the plastid in malaria parasites. Mol Microbiol 61: 614–630. 16787449 10.1111/j.1365-2958.2006.05244.x

[13] van Dooren GG, Tomova C, Agrawal S, Humbel BM, Striepen B (2008) *Toxoplasma gondii* Tic20 is essential for apicoplast protein import. Proc Natl Acad Sci USA 105: 13574–13579. 18757752 10.1073/pnas.0803862105 PMC2533231

[14] Kalanon M, Tonkin CJ, McFadden GI (2009) Characterization of two putative protein translocation components in the apicoplast of *Plasmodium falciparum* . Eukaryot Cell 8: 1146–1154. 19502580 10.1128/EC.00061-09 PMC2725556

[15] Glaser S, van Dooren GG, Agrawal S, Brooks CF, McFadden GI, et al (2012) Tic22 is an essential chaperone required for protein import into the apicoplast. J Biol Chem 287: 39505–39512. 23027875 10.1074/jbc.M112.405100 PMC3501059

[16] Mullin KA, Lim L, Ralph SA, Spurck TP, Handman E, et al (2006) Membrane transporters in the relict plastid of malaria parasites. Proc Natl Acad Sci USA 103: 9572–9577. 16760253 10.1073/pnas.0602293103 PMC1480448

[17] Sommer MS, Gould SB, Lehmann P, Gruber A, Przyborski JM, et al (2007) Der1-mediated pre-protein import into the periplastid compartment of chromalveolates? Mol Biol Evol 24: 918–928. 17244602 10.1093/molbev/msm008

[18] Spork S, Hiss JA, Mandel K, Sommer M, Kooij TW, et al (2009) An unusual ERAD-like complex is targeted to the apicoplast of *Plasmodium falciparum* . Eukaryot Cell 8: 1134–1145. 19502583 10.1128/EC.00083-09 PMC2725561

[19] Agrawal S, van Dooren GG, Beatty WL, Striepen B (2009) Genetic evidence that an endosymbiont-derived ERAD system functions in import of apicoplast proteins. J Biol Chem 284: 33683–33691. 19808683 10.1074/jbc.M109.044024 PMC2785210

[20] Sheiner L, Demerly JL, Poulsen N, Beatty WL, Lucas O, et al (2011) A systematic screen to discover and analyze apicoplast proteins identifies a conserved and essential protein import factor. PLoS Pathog 7: e1002392. 22144892 10.1371/journal.ppat.1002392 PMC3228799

[21] Karnataki A, DeRocher AE, Feagin JE, Parsons M (2009) Sequential processing of the *Toxoplasma* apicoplast membrane protein FtsH1 in topologically distinct domains during intracellular trafficking. Mol Biochem Parasitol 166: 126–133. 19450729 10.1016/j.molbiopara.2009.03.004 PMC2817949

[22] Karnataki A, DeRocher AE, Coppens I, Feagin JE, Parsons M (2007) A membrane protease is targeted to the relict plastid of *Toxoplasma* via an internal signal sequence. Traffic 8: 1543–1553. 17822404 10.1111/j.1600-0854.2007.00637.x

[23] DeRocher AE, Coppens I, Karnataki A, Gilbert LA, Rome ME, et al (2008) A thioredoxin family protein of the apicoplast periphery identifies abundant candidate transport vesicles in *Toxoplasma gondii* . Eukaryot Cell 7: 1518–1529. 18586952 10.1128/EC.00081-08 PMC2547066

[24] Karnataki A, DeRocher A, Coppens I, Nash C, Feagin JE, et al (2007) Cell cycle-regulated vesicular trafficking of *Toxoplasma* APT1, a protein localized to multiple apicoplast membranes. Mol Microbiol 63: 1653–1668. 17367386 10.1111/j.1365-2958.2007.05619.x

[25] DeRocher AE, Karnataki A, Vaney P, Parsons M (2012) Apicoplast targeting of a *T. gondii* transmembrane protein requires a cytosolic tyrosine-based motif. Traffic 13: 694–704. 22288938 10.1111/j.1600-0854.2012.01335.x PMC3324616

[26] Striepen B, Crawford MJ, Shaw MK, Tilney LG, Seeber F, et al (2000) The plastid of *Toxoplasma gondii* is divided by association with the centrosomes. J Cell Biol 151: 1423–1434. 11134072 10.1083/jcb.151.7.1423 PMC2150670

[27] Tawk L, Dubremetz JF, Montcourrier P, Chicanne G, Merezegue F, et al (2011) Phosphatidylinositol 3-monophosphate is involved in *Toxoplasma* apicoplast biogenesis. PLoS Pathog 7: e1001286. 21379336 10.1371/journal.ppat.1001286 PMC3040667

[28] Behnke MS, Wootton JC, Lehmann MM, Radke JB, Lucas O, et al (2010) Coordinated progression through two subtranscriptomes underlies the tachyzoite cycle of *Toxoplasma gondii* . PLoS ONE 5: e12354. 20865045 10.1371/journal.pone.0012354 PMC2928733

[29] Gurskaya NG, Fradkov AF, Terskikh A, Matz MV, Labas YA, et al (2001) GFP-like chromoproteins as a source of far-red fluorescent proteins. FEBS Lett 507: 16–20. 11682051 10.1016/s0014-5793(01)02930-1

[30] He CY, Shaw MK, Pletcher CH, Striepen B, Tilney LG, et al (2001) A plastid segregation defect in the protozoan parasite *Toxoplasma gondii* . EMBO J 20: 330–339. 11157740 10.1093/emboj/20.3.330 PMC133478

[31] Harper JM, Huynh MH, Coppens I, Parussini F, Moreno S, et al (2006) A cleavable propeptide influences *Toxoplasma* infection by facilitating the trafficking and secretion of the TgMIC2-M2AP invasion complex. Mol Biol Cell 17: 4551–4563. 16914527 10.1091/mbc.E06-01-0064 PMC1635346

[32] Pelletier L, Stern CA, Pypaert M, Sheff D, Ngo HM, et al (2002) Golgi biogenesis in *Toxoplasma gondii* . Nature 418: 548–552. 12152082 10.1038/nature00946

[33] Kohler S (2005) Multi-membrane-bound structures of Apicomplexa: I. the architecture of the *Toxoplasma gondii* apicoplast. Parasitol Res 96: 258–272. 15895255 10.1007/s00436-005-1338-2

[34] Caffaro CE, Koshy AA, Liu L, Zeiner GM, Hirschberg CB, et al (2013) A nucleotide sugar transporter involved in glycosylation of the toxoplasma tissue cyst wall is required for efficient persistence of bradyzoites. PLoS Pathog 9: e1003331. 23658519 10.1371/journal.ppat.1003331 PMC3642066

[35] Doms RW, Russ G, Yewdell JW (1989) Brefeldin A redistributes resident and itinerant Golgi proteins to the endoplasmic reticulum. J Cell Biol 109: 61–72. 2745557 10.1083/jcb.109.1.61 PMC2115463

[36] Sato K, Nakano A (2005) Dissection of COPII subunit-cargo assembly and disassembly kinetics during Sar1p-GTP hydrolysis. Nat Struct Mol Biol 12: 167–174. 15665868 10.1038/nsmb893

[37] Gorelick FS, Shugrue C (2001) Exiting the endoplasmic reticulum. Mol Cell Endocrinol 177: 13–18. 11377815 10.1016/s0303-7207(01)00438-5

[38] Nakano A, Muramatsu M (1989) A novel GTP-binding protein, Sar1p, is involved in transport from the endoplasmic reticulum to the Golgi apparatus. J Cell Biol 109: 2677–2691. 2512296 10.1083/jcb.109.6.2677 PMC2115904

[39] Barlowe C, Orci L, Yeung T, Hosobuchi M, Hamamoto S, et al (1994) COPII: a membrane coat formed by Sec proteins that drive vesicle budding from the endoplasmic reticulum. Cell 77: 895–907. 8004676 10.1016/0092-8674(94)90138-4

[40] Forster R, Weiss M, Zimmermann T, Reynaud EG, Verissimo F, et al (2006) Secretory cargo regulates the turnover of COPII subunits at single ER exit sites. Curr Biol 16: 173–179. 16431369 10.1016/j.cub.2005.11.076

[41] Lee MC, Orci L, Hamamoto S, Futai E, Ravazzola M, et al (2005) Sar1p N-terminal helix initiates membrane curvature and completes the fission of a COPII vesicle. Cell 122: 605–617. 16122427 10.1016/j.cell.2005.07.025

[42] Weissman JT, Plutner H, Balch WE (2001) The mammalian guanine nucleotide exchange factor mSec12 is essential for activation of the Sar1 GTPase directing endoplasmic reticulum export. Traffic 2: 465–475. 11422940 10.1034/j.1600-0854.2001.20704.x

[43] Kuge O, Dascher C, Orci L, Rowe T, Amherdt M, et al (1994) Sar1 promotes vesicle budding from the endoplasmic reticulum but not Golgi compartments. J Cell Biol 125: 51–65. 8138575 10.1083/jcb.125.1.51 PMC2120015

[44] Adisa A, Frankland S, Rug M, Jackson K, Maier AG, et al (2007) Re-assessing the locations of components of the classical vesicle-mediated trafficking machinery in transfected *Plasmodium falciparum* . Int J Parasitol 37: 1127–1141. 17428488 10.1016/j.ijpara.2007.02.009

[45] Nakano A, Otsuka H, Yamagishi M, Yamamoto E, Kimura K, et al (1994) Mutational analysis of the Sar1 protein, a small GTPase which is essential for vesicular transport from the endoplasmic reticulum. J Biochem 116: 243–247. 7822237 10.1093/oxfordjournals.jbchem.a124513

[46] Bangs JD, Uyetake L, Brickman MJ, Balber AE, Boothroyd JC (1993) Molecular cloning and cellular localization of a BiP homologue in *Trypanosoma brucei*. Divergent ER retention signals in a lower eukaryote. J Cell Sci 105: 1101–1113. 8227199 10.1242/jcs.105.4.1101

[47] Ward TH, Polishchuk RS, Caplan S, Hirschberg K, Lippincott-Schwartz J (2001) Maintenance of Golgi structure and function depends on the integrity of ER export. J Cell Biol 155: 557–570. 11706049 10.1083/jcb.200107045 PMC2198855

[48] Jullien N, Sampieri F, Enjalbert A, Herman JP (2003) Regulation of Cre recombinase by ligand-induced complementation of inactive fragments. Nucleic Acids Res 31: e131. 14576331 10.1093/nar/gng131 PMC275488

[49] Andenmatten N, Egarter S, Jackson AJ, Jullien N, Herman JP, et al (2013) Conditional genome engineering in *Toxoplasma gondii* uncovers alternative invasion mechanisms. Nat Methods 10: 125–127. 23263690 10.1038/nmeth.2301 PMC3605914

[50] Farese RV Jr, Walther TC (2009) Lipid droplets finally get a little R-E-S-P-E-C-T. Cell 139: 855–860. 19945371 10.1016/j.cell.2009.11.005 PMC3097139

[51] Guo Y, Cordes KR, Farese RV Jr, Walther TC (2009) Lipid droplets at a glance. J Cell Sci 122: 749–752. 19261844 10.1242/jcs.037630 PMC2714424

[52] Sulli C, Schwartzbach SD (1995) The polyprotein precursor to the *Euglena* light-harvesting chlorophyl a/b-binding protein is transported to the Golgi apparatus prior to chloroplast import and polyprotein processing. J Biol Chem 270: 13084–13090. 7768903 10.1074/jbc.270.22.13084

[53] Sulli C, Fang Z, Muchhal U, Schwartzbach SD (1999) Topology of *Euglena* chloroplast protein precursors within endoplasmic reticulum to Golgi to chloroplast transport vesicles. J Biol Chem 274: 457–463. 9867865 10.1074/jbc.274.1.457

[54] Nassoury N, Cappadocia M, Morse D (2003) Plastid ultrastructure defines the protein import pathway in dinoflagellates. J Cell Sci 116: 2867–2874. 12771189 10.1242/jcs.00517

[55] Chaudhari R, Narayan A, Patankar S (2012) A novel trafficking pathway in *Plasmodium falciparum* for the organellar localization of glutathione peroxidase-like thioredoxin peroxidase. FEBS J 279: 3872–3888. 22889167 10.1111/j.1742-4658.2012.08746.x

[56] Kilian O, Kroth PG (2005) Identification and characterization of a new conserved motif within the presequence of proteins targeted into complex diatom plastids. Plant J 41: 175–183. 15634195 10.1111/j.1365-313X.2004.02294.x

[57] Coppens I, Joiner KA (2003) Host but not parasite cholesterol controls *Toxoplasma* cell entry by modulating organelle discharge. Mol Biol Cell 14: 3804–3820. 12972565 10.1091/mbc.E02-12-0830 PMC196568

[58] Coppens I (2006) Contribution of host lipids to *Toxoplasma* pathogenesis. Cell Microbiol 8: 1–9. 16367861 10.1111/j.1462-5822.2005.00647.x

[59] Ramakrishnan S, Docampo MD, Macrae JI, Pujol FM, Brooks CF, et al (2012) The apicoplast and endoplasmic reticulum cooperate in fatty acid biosynthesis in the apicomplexan parasite Toxoplasma gondii. J Biol Chem 287: 4957–4971. 22179608 10.1074/jbc.M111.310144 PMC3281623

[60] Li-Beisson Y, Shorrosh B, Beisson F, Andersson MX, Arondel V, et al (2013) Acyl-lipid metabolism. Arabidopsis Book 11: e0161. 23505340 10.1199/tab.0161 PMC3563272

[61] Schnurr JA, Shockey JM, De Boer GJ, Browse JA (2002) Fatty Acid export from the chloroplast. Molecular characterization of a major plastidial acyl-coenzyme a synthetase from *Arabidopsis* . Plant Physiol 129: 1700–1709. 12177483 10.1104/pp.003251 PMC166758

[62] Wang Z, Benning C (2012) Chloroplast lipid synthesis and lipid trafficking through ER-plastid membrane contact sites. Biochem Soc Trans 40: 457–463. 22435830 10.1042/BST20110752

[63] Meissner M, Brecht S, Bujard H, Soldati D (2001) Modulation of myosin A expression by a newly established tetracycline repressor-based inducible system in *Toxoplasma gondii* . Nucleic Acids Res 29: E115. 11713335 10.1093/nar/29.22.e115 PMC92585

[64] van Poppel NF, Welagen J, Duisters RF, Vermeulen AN, Schaap D (2006) Tight control of transcription in *Toxoplasma gondii* using an alternative tet repressor. Int J Parasitol 36: 443–452. 16516216 10.1016/j.ijpara.2006.01.005

[65] Herm-Gotz A, Agop-Nersesian C, Munter S, Grimley JS, Wandless TJ, et al (2007) Rapid control of protein level in the apicomplexan *Toxoplasma gondii* . Nat Methods 4: 1003–1005. 17994029 10.1038/nmeth1134 PMC2601725

[66] Tsien JZ, Chen DF, Gerber D, Tom C, Mercer EH, et al (1996) Subregion- and cell type-restricted gene knockout in mouse brain. Cell 87: 1317–1326. 8980237 10.1016/s0092-8674(00)81826-7

[67] Daher JP, Ying M, Banerjee R, McDonald RS, Hahn MD, et al (2009) Conditional transgenic mice expressing C-terminally truncated human alpha-synuclein (alphaSyn119) exhibit reduced striatal dopamine without loss of nigrostriatal pathway dopaminergic neurons. Mol Neurodegener 4: 34. 19630976 10.1186/1750-1326-4-34 PMC2722624

[68] Donald RK, Carter D, Ullman B, Roos DS (1996) Insertional tagging, cloning, and expression of the *Toxoplasma gondii* hypoxanthine-xanthine-guanine phosphoribosyltransferase gene. Use as a selectable marker for stable transformation. J Biol Chem 271: 14010–14019. 8662859 10.1074/jbc.271.24.14010

[69] Shorter J, Watson R, Giannakou ME, Clarke M, Warren G, et al (1999) GRASP55, a second mammalian GRASP protein involved in the stacking of Golgi cisternae in a cell-free system. EMBO J 18: 4949–4960. 10487747 10.1093/emboj/18.18.4949 PMC1171566

[70] DeRocher A, Hagen CB, Froehlich JE, Feagin JE, Parsons M (2000) Analysis of targeting sequences demonstrates that trafficking to the *Toxoplasma gondii* plastid branches off the secretory system. J Cell Sci 113: 3969–3977. 11058084 10.1242/jcs.113.22.3969

[71] He CY, Striepen B, Pletcher CH, Murray JM, Roos DS (2001) Targeting and processing of nuclear-encoded apicoplast proteins in plastid segregation mutants of *Toxoplasma gondii* . J Biol Chem 276: 28436–28442. 11319231 10.1074/jbc.M102000200

[72] Bradley PJ, Ward C, Cheng SJ, Alexander DL, Coller S, et al (2005) Proteomic analysis of rhoptry organelles reveals many novel constituents for host-parasite interactions in *Toxoplasma gondii* . J Biol Chem 280: 34245–34258. 16002398 10.1074/jbc.M504158200

[73] Hehl AB, Lekutis C, Grigg ME, Bradley PJ, Dubremetz JF, et al (2000) *Toxoplasma gondii* homologue of plasmodium apical membrane antigen 1 is involved in invasion of host cells. Infect Immun 68: 7078–7086. 11083833 10.1128/iai.68.12.7078-7086.2000 PMC97818

[74] Jelenska J, Crawford MJ, Harb OS, Zuther E, Haselkorn R, et al (2001) Subcellular localization of acetyl-CoA carboxylase in the apicomplexan parasite *Toxoplasma gondii* . Proc Natl Acad Sci USA 98: 2723–2728. 11226307 10.1073/pnas.051629998 PMC30206

[75] Brydges SD, Sherman GD, Nockemann S, Loyens A, Daubener W, et al (2000) Molecular characterization of TgMIC5, a proteolytically processed antigen secreted from the micronemes of *Toxoplasma gondii* . Mol Biochem Parasitol 111: 51–66. 11087916 10.1016/s0166-6851(00)00296-6

