# Supplementary figures and images for: Vesicles Bearing Toxoplasma Apicoplast Membrane Proteins Persist Following Loss of the Relict Plastid or Golgi Body Disruption
Source: PLoS One. 2014 Nov 4;9(11):e112096. doi: 10.1371/journal.pone.0112096 (PMC4219833; doi:10.1371/journal.pone.0112096)

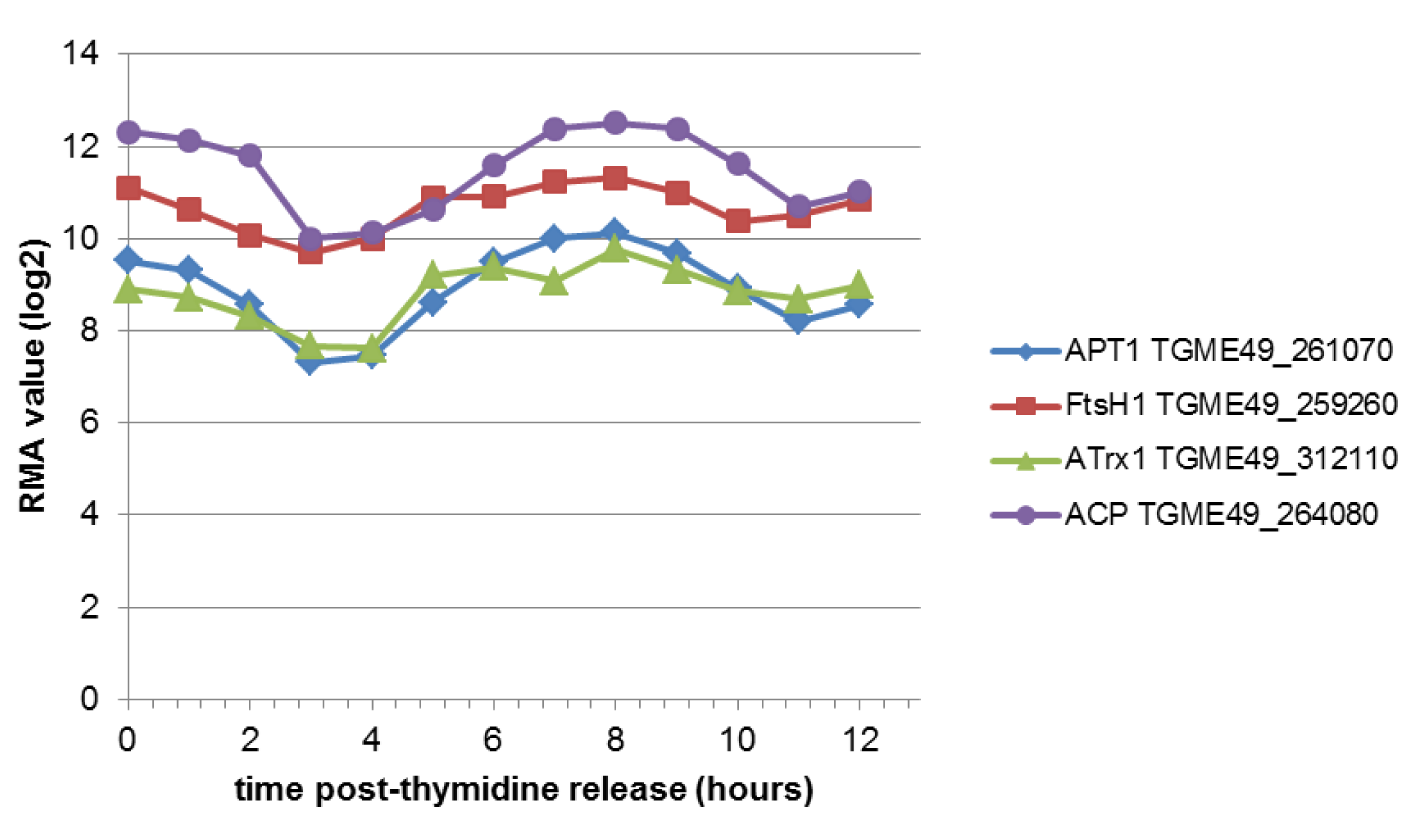

Supplement: Figure S1 — Cell cycle regulation of transcription of genes in this study. Quantitation of expression of the relevant genes following post-thymidine block release of T. gondii RHTK+ parasites was obtained from ToxoDB, based on microarray data from [28]. The time period covers approximately 1.66 cell cycles, with internal daughter cells peaking at 4 and 12 hours. These data show that the promoters for apicoplast reporters utilized in this study have very similar temporal kinetics. (TIF) [file pone.0112096.s001.tif]

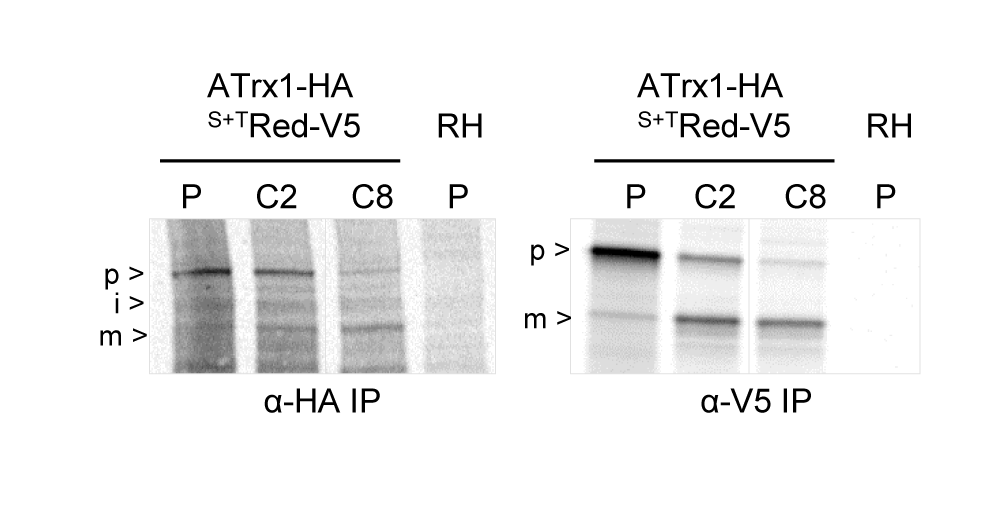

Supplement: Figure S2 — Similar half-life of S+TRed-V5 and ATrx1. Pulse-chase analysis was carried out as in Fig. 6, with S+TRed-V5 and ATrx1-HA co-expressed in the same parasite line. The molecules were sequentially immunoprecipitated with mAbs directed against the epitope tags and 35S-methionine labeled proteins detected by phosphorimaging. For each antibody, the lanes shown are from the same scan of the gel. Three main bands are seen for ATrx1-HA, with the 90 kDa protein being a precursor (p) to intermediate (i) and mature 65 kDa protein (m) [23]. The subcellular location where processing occurs is not known. The cleavage of the precursor (p) S+TRed-V5 to mature form (m, 35 kDa) occurs within the apicoplast. (TIF) [file pone.0112096.s002.tif]

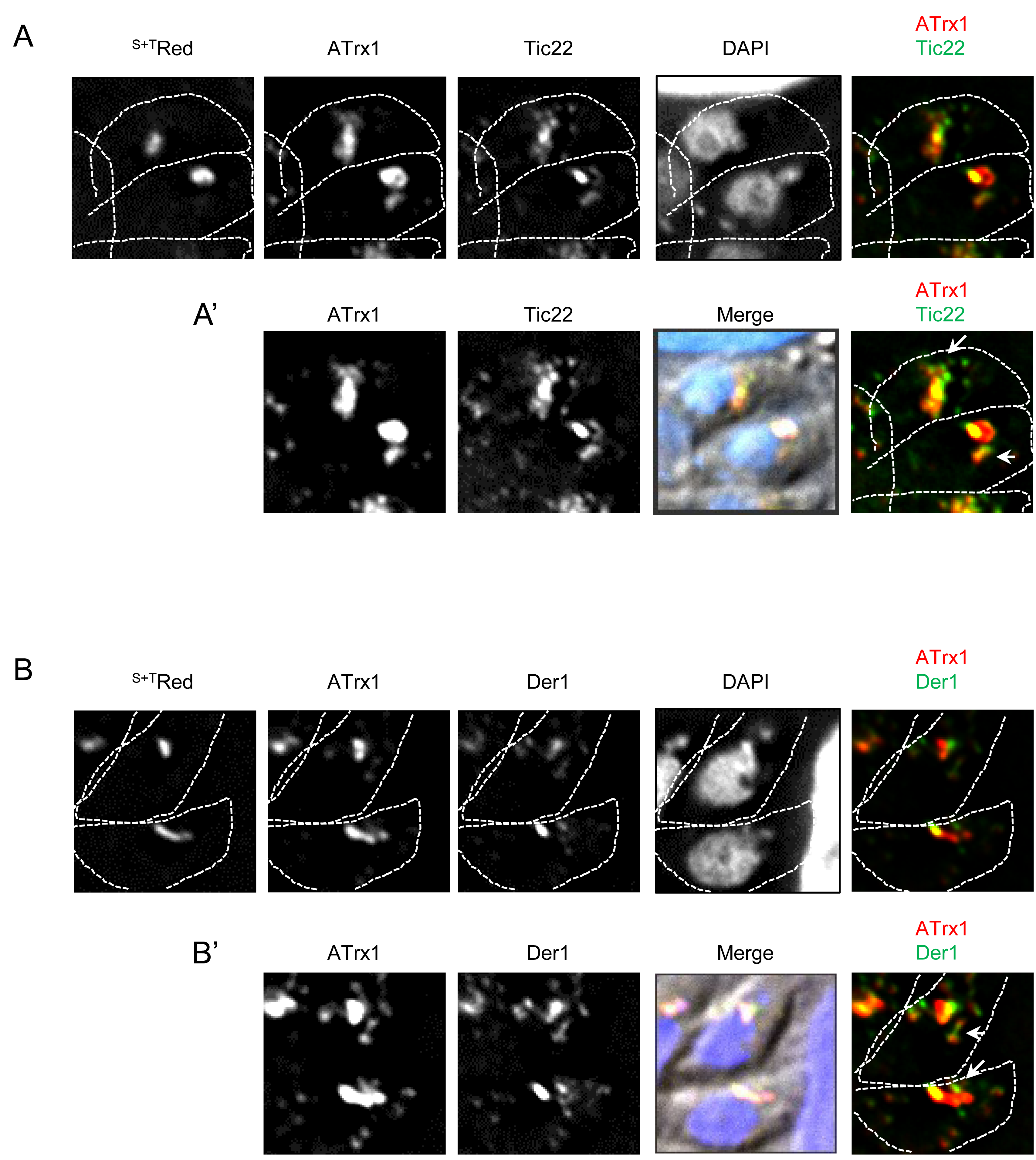

Supplement: Figure S3 — Tic22 and Der1ap inhabit Vap. Clonal lines expressing the apicoplast luminal marker S+TRed and either Tic22-HA or Der1-HA T. gondii within fibroblasts were processed for IFA and stained for ATrx1 (using mAb 11G8 followed by anti-mouse IgG coupled to DyLight 649) and for Tic22-HA or Der1-HA (using rat anti-HA mAb coupled to FITC). Slides were co-stained with DAPI. The apicoplast luminal marker S+TRed was detected by endogenous fluorescence. Dotted lines indicate outline of parasites within vacuole. A) Localization of Tic22-HA. Red, ATrx1; green, Tic22-HA. Panel A′ shows images with enhanced scaling to reveal Vap and a merge image showing DIC (grey), DAPI (blue) Tic22 (green) S+TRed (orange) and ATrx1 (maroon). Arrows indicate Vap containing both Tic22 and ATrx1. B) Localization of Der1ap-HA. Red, ATrx1; green, Der1-HA. Panel B′ shows images with enhanced scaling to reveal Vap and a merge image showing DIC (grey), DAPI (blue) Der1 (green) S+TRed (orange) and ATrx1 (maroon). Arrows indicate Vap containing both Der1 and ATrx1. (TIF) [file pone.0112096.s003.tif]

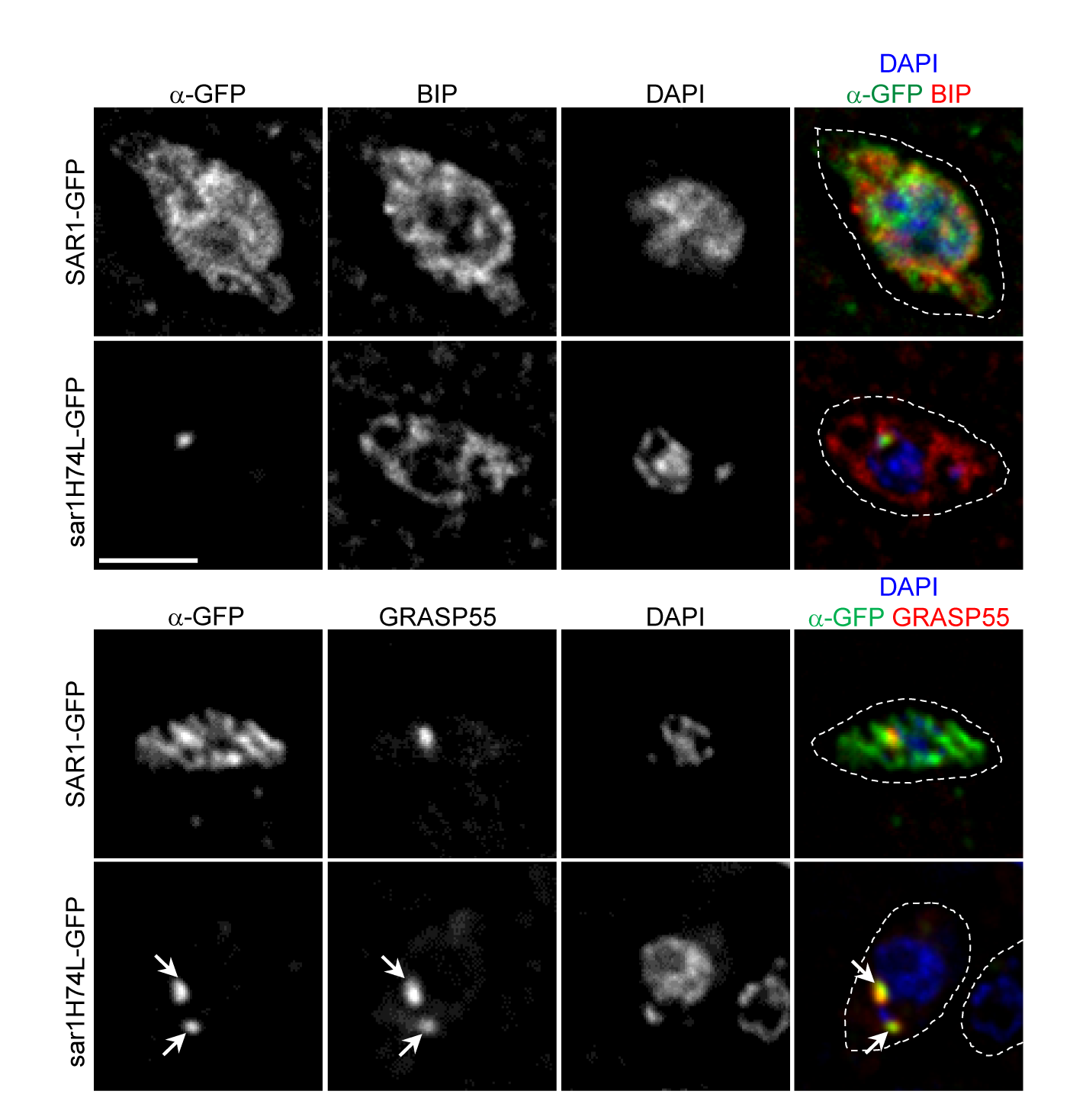

Supplement: Figure S4 — Localization of SAR1-GFP and sar1(H74L)-GFP in T. gondii . The constructs were transiently transfected into T. gondii expressing GRASP55-HcRed. After 11 hours, the samples were fixed and subjected to IFA, comparing the localization of SAR1 and sar1(H74L) (endogenous green fluorescence) to GRASP55 and BiP, an ER marker protein detected anti-T. brucei BiP followed by anti-rabbit IgG coupled to Alexa 680. Arrow marks colocalization of sar1(H74L)-GFP and GRASP55. This particular cell has duplicated its Golgi body. “H” marks a host cell nucleus. Bar, 2 µM. (TIF) [file pone.0112096.s004.tif]

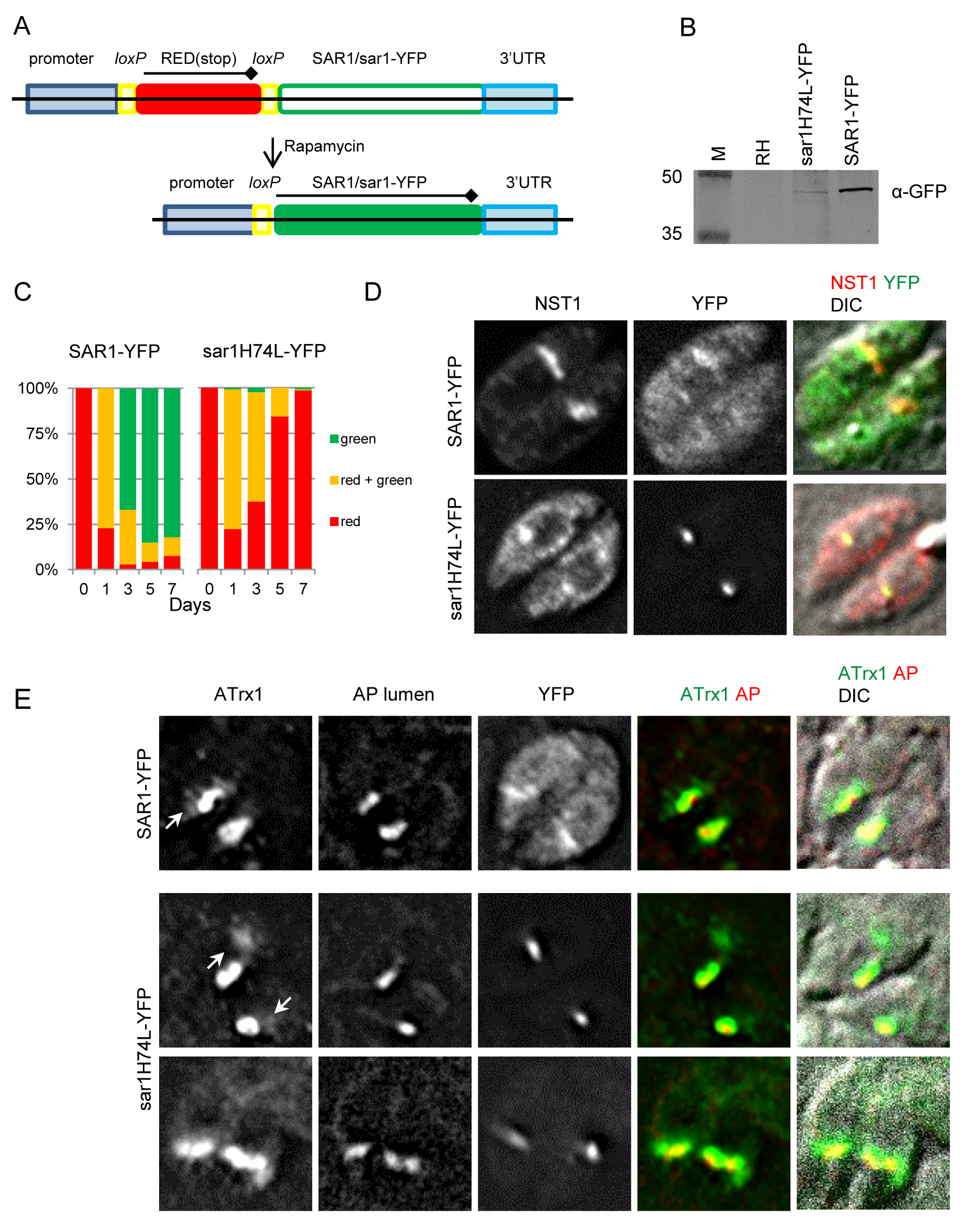

Supplement: Figure S5 — Conditional expression of sar1(H74L)-YFP in T. gondii . A) Map of expression locus before (top) and after (bottom) excision of the loxP-flanked red fluorescent protein coding sequence which separates the promoter from the SAR1-YFP fusion proteins. Translated segments are indicated by dark fill with a line above.B) Western blot of protein from the parental RH parasites, and 24 hour rapamycin induced sar1(H74L)-YFP and SAR1-YFP parasites probed with anti-GFP. M, markers. The fusion proteins migrated at the expected size (49 kDa). C) Parasites expressing sar1(H74L) are lost upon cultivation. After rapamycin mediated induction of expression (via excision of the RFP gene), the percentage of vacuoles in each population with parasites expressing YFP-tagged SAR1 or sar1(H74L) was monitored over time. All parasites in a given vacuole showed the same expression phenotype. Before excision (day 0) both parasite lines showed red fluorescence only. For an intermediate period many parasites expressed both yellow fluorescent protein and previously transcribed and translated red fluorescent protein. Those parasites in which expression of SAR1-YFP was induced continue to grow and became YFP+/RFP−, whereas those expressing sar1(H74L)-YFP did not survive and were outgrown by the minority population that had not excised the RFP coding sequence (n>200 vacuoles for each time point). D) The conditionally expressed mutant sar1(H74L) disrupts the Golgi body. The SAR1/sar1 clonal parasite lines were transiently transfected with NST1-HA and after 15 hours rapamycin was added. Parasites were analyzed 11 hours later and representative examples are shown. Blind analysis indicated that NST1-HA was localized to the Golgi body in 95% of parasites expressing SAR1-YFP, but was redistributed to the ER in 81% of those parasites expressing sar1(H74L). E) Vap persist in parasites expressing dominant negative sar1(H74L). Expression of wt or mutant SAR1 was induced by the addition of rapamycin and after 11 [file pone.0112096.s005.tif]
